# MR molecular imaging for monitoring and predicting tumor responses to immunotherapy

**DOI:** 10.7150/thno.106481

**Published:** 2025-09-22

**Authors:** Victoria E. A. Laney, Walter Zhao, Inga M. Hwang, Emma Hampson, Juan Dong, Ryan C. Hall, Kristin Weber-Bonk, Xueer Yuan, Elizabeth Delaney, Hannah Gilmore, Ruth Keri, Jordan Winter, Li Lily Wang, Zheng-Rong Lu

**Affiliations:** 1 Department of Biomedical Engineering, Case Western Reserve University, Cleveland, OH 44106, USA.; 2 Medical Scientist Training Program, Case Western Reserve University School of Medicine, Cleveland, OH 44106, USA.; 3 Department of Translational Hematology and Oncology Research, Lerner Research Institute, Cleveland Clinic Foundation, Cleveland, OH 44106, USA.; 4 Department of Cancer Biology, Lerner Research Institute, Cleveland Clinic Foundation, Cleveland, OH 44195, USA.; 5 Department of Pathology, Cleveland Clinic Foundation, Cleveland, OH, 44195, USA.; 6 Case Comprehensive Cancer Center, Case Western Reserve University, Cleveland, OH 44106, USA.; 7 Department of Surgery, University Hospitals Cleveland Medical Center, Cleveland, OH 44106, USA.

**Keywords:** MR molecular imaging, targeted MRI contrast agent, MT218, cancer immunotherapy, extradomain B fibronectin, therapeutic response

## Abstract

**Background:** While immunotherapies show great promise in cancer treatment, variability in patient responses warrant the need for improved methods to assess early responses and guide precision therapy. The tumor microenvironment (TME) plays a critical role in antitumor immunity and tumor response to immunotherapy. Critically, TME components and their crosstalk with immune cells can be leveraged as a prognostic marker for therapeutic response. This study evaluated the use of magnetic resonance molecular imaging (MRMI) targeting the TME protein extradomain B fibronectin (EDB-FN), which is a lymphokine secreted by activated T lymphocytes and a marker of the epithelial-to-mesenchymal transition (EMT) in aggressive tumor cells. MRMI of EDB-FN was evaluated within the tumor extracellular matrix and was correlated with immunotherapy-related outcomes.

**Methods:** C57BL/6 mice bearing orthotopic KPC pancreatic tumors were treated with a novel immune checkpoint inhibitor (VISTA-blocking antibodies), a vaccine (mutant KRAS^G12D^ peptide with TLR7/8/9 agonists), or a combination of both. MRMI with an EDB-FN-specific contrast agent, MT218, was used to image tumor responses during treatment. T_1_-weighted MRI (fast spin echo and FLASH sequences) was acquired before, during, and after tumor treatment. Tumor signal enhancement patterns were analyzed to assess treatment response. EDB-FN expression and the infiltration of CD4^+^ and CD8^+^ T lymphocytes in the tumors were determined by immunohistochemistry (IHC) and immunofluorescence (IF) staining, respectively, and correlated with MRMI observations, tumor response, and therapeutic outcomes.

**Results:** MT218-MRMI revealed distinctive signal enhancement patterns across different treatments. These patterns were detected as early as two weeks after treatment initiation and correlated strongly with EDB-FN expression and CD4^+^ and CD8^+^ T cell infiltration, as confirmed by IHC and IF. Signal profiles corresponded to known TME phenotypes: immune desert, immune excluded, and immune inflamed, which were associated with non-response, partial response, and complete response, respectively. By four weeks post-treatment, MRMI criteria successfully distinguished complete responders from partial responders. Over a 200-day monitoring period, outcome prediction showed complete (100%) long-term disease-free survival in complete responders, 24-27% survival in partial responders, and no (0%) survival in non-responders and those with stable disease.

**Conclusion:** MT218-MRMI non-invasively distinguishes tumor response patterns with significant potential for early prediction of therapeutic outcomes and timely optimization of treatment strategies. While further validation is needed for clinical translation, these findings demonstrate MT218-MRMI's promise as a tool for monitoring immunotherapy response in pancreatic cancer and underscore its potential utility for precision immunotherapy.

## Introduction

Immunotherapy has revolutionized cancer treatment and improved survival outcomes across multiple tumor types 1. In addition to immune checkpoint inhibitors, anti-tumor vaccines, such as cancer-specific mRNA and neoantigen vaccines, have demonstrated a synergistic effect when combined with other immunotherapies 2-6. However, individual patient responses remain variable, with suboptimal therapeutic outcomes in many cancers 7. Current diagnostic tools lack sufficient accuracy to reliably distinguish responders from non-responders and predict treatment outcome. As a result, non-responders often endure treatment-related side effects without therapeutic benefit and may lose opportunities to pursue alternative treatments. Therefore, there is a critical need to develop non-invasive, accurate, and accessible tools to monitor and predict tumor responses to immunotherapies.

Effective immunotherapies rely on activation and recruitment of helper and cytotoxic T-lymphocytes into cancerous tissues 2. Impaired T cell response to immunotherapy may result from insufficient infiltration and dysfunction of cytotoxic T lymphocytes. Beyond programmed death-1 (PD-1) and cytotoxic T-lymphocyte-associated protein 4 (CTLA-4), novel immune checkpoint receptors (ICRs) play non-redundant roles in suppressing anti-tumor T cell responses. Among these, the V-domain immunoglobulin suppressor of T cell activation (VISTA, gene *Vsir*) is a highly promising therapeutic target 8-10. Previous studies have established that VISTA regulates anti-tumor immunity through multiple mechanisms, including promoting the metabolism and differentiation of myeloid-derived suppressor cells 11, impairing toll-like receptor (TLR) signaling and macrophage activation 12, and inhibiting anti-tumor T cell activation through cell surface protein LRIG1 engagement 8, 9, 13-17. VISTA blockade has been shown to reduce tumor growth in several preclinical tumor models 14, 15, 18, 19, while in human cancers VISTA expression on tumor-infiltrating myeloid cells and T-cells has been associated with therapeutic resistance and recurrence 9, 20-23.

The tumor microenvironment (TME) plays a crucial role in anti-cancer immunity. The TME consists of extracellular matrix, stromal cells, and immune cells, and regulates infiltration of cytotoxic T-lymphocytes, which are critical players in immunotherapeutic responses. The TME exhibits three distinct phenotypes distinguished by immune cell presence and distribution: immune desert (absent cytotoxic T lymphocytes), immune excluded (peripheral presence without infiltration), and immune inflamed (substantial infiltration). These phenotypes strongly correlate with immunotherapy response outcomes 26, 27. Molecular imaging of TME immune phenotypes thus has the potential to non-invasively monitor tumor response and predict therapeutic outcomes 28, 29.

Within the TME, the ECM orchestrates immune cell activation, differentiation, and infiltration 24, 25. Fibronectin (FN), a key acellular component of the ECM, plays a crucial role in ECM assembly and organization by modulating cell adhesion, proliferation, and migration 30, 31. As a marker of inflammation, tissue remodeling, and the epithelial-mesenchymal transition (EMT) 32-35, FN also regulates T lymphocyte development, activation, migration, and proliferation 31, 36-40. Importantly, an oncofetal subtype of FN called extradomain B fibronectin isoform (EDB-FN) is selectively overexpressed in aggressive human cancers (including pancreatic cancer) as shown by RNA-seq analysis and immunohistochemistry (IHC) 41-43. EDB-FN is a valuable imaging target because it is overexpressed in the tumor ECM and correlates strongly with tumor grade and stage, while being minimally present in normal tissue 41, 42. Moreover, EDB-FN is a lymphokine secreted by activated T cells (CD4⁺ and CD8⁺) 44, 45, further elevating its attractiveness as an ideal marker for real-time assessment of immunotherapy responses. While various EDB-FN molecular imaging agents for cancer detection and diagnosis 46-49 have been developed, EDB-FN's potential for immune activation monitoring has not been explored.

**Figure 1:**
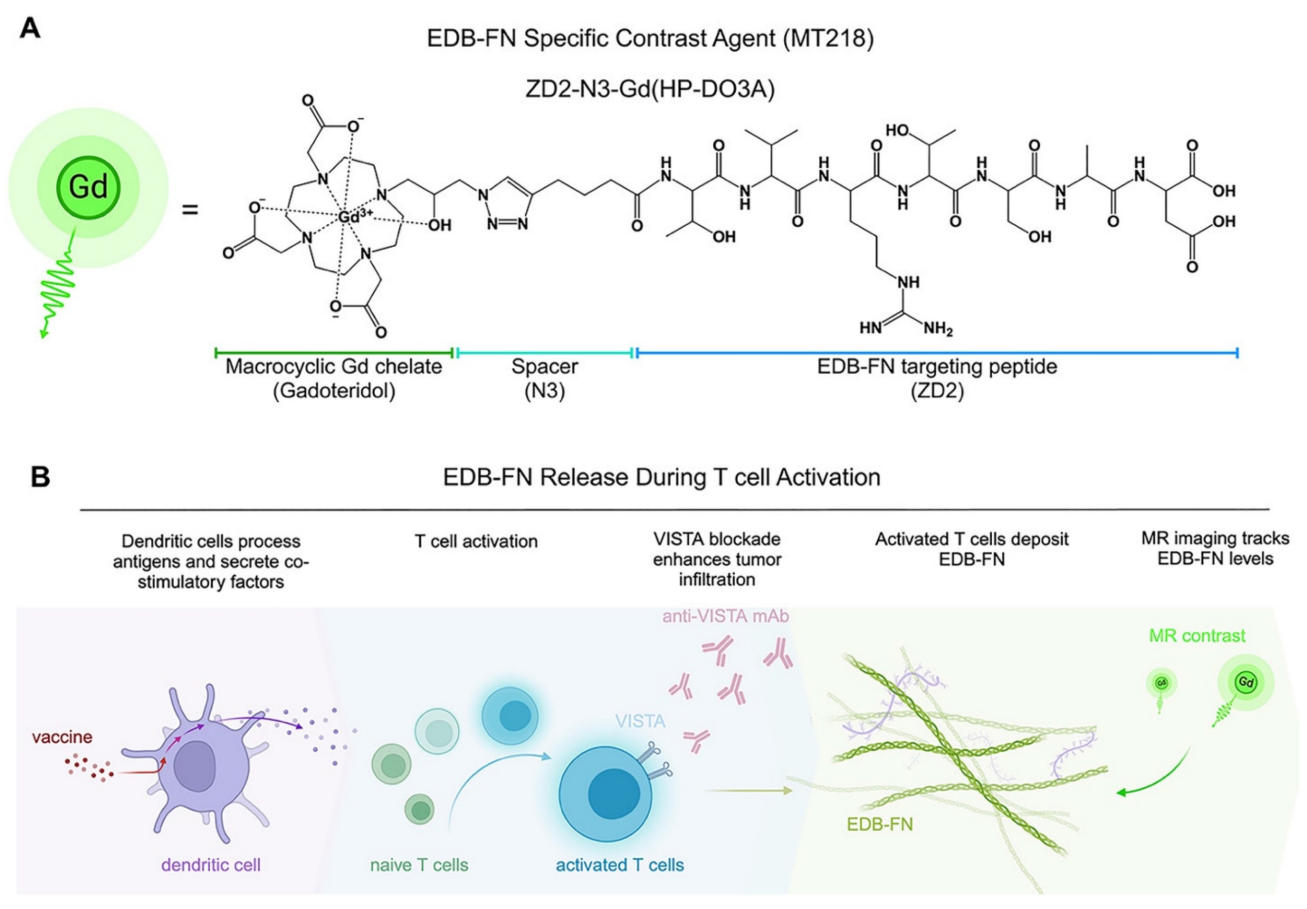
** Conceptual overview of MT218-MRMI and its application to PDAC immunotherapy. A)** Chemical structure of the EDB-FN-specific contrast agent, MT218 (right), alongside its schematic representation (left). **B)** Immunotherapy with peptide vaccines and anti-VISTA antibodies activates T cells, which subsequently promote EDB-FN secretion and deposition as part of immune-stromal crosstalk. MT218-MRMI enables visualization of EDB-FN expression through contrast enhancement, allowing for non-invasive monitoring of dynamic TME changes throughout the course of immunotherapy.

MRI is a clinical imaging modality and provides three-dimensional images of soft tissues, including tumors, with high spatial resolution. It is routinely used for cancer diagnosis, treatment planning, and image-aided precision cancer therapy, including immunotherapy. However, the potential of MRI is limited by the lack of a targeted contrast agent that can characterize tumor responses to immunotherapies. We have developed a small peptide targeted MRI contrast agent, ZD2-N3-Gd(HP-DO3A) or MT218, specific to EDB-FN for magnetic resonance molecular imaging (MRMI) of cancer 50. MT218 is a targeted contrast agent consisting of a small peptide ZD2 (Thr-Val-Arg-Thr-Ser-Ala-Asp) conjugated with gadoteridol that specifically binds the EDB fragment (**Figure 1A**) 51. As shown in previous studies, EDB-FN is overexpressed in human pancreatic ductal adenocarcinoma (PDAC) tumors 41, 43. MRMI with MT218 (MT218-MRMI) effectively detects EDB-FN changes in multiple aggressive cancers, including breast, colorectal, head and neck, lung, pancreatic and prostate 52, 53. In murine models, we have also shown that MT218-MRMI can monitor tumor progression and therapeutic response to conventional therapies 54-56. These results have led to an FDA-approved clinical trial (NCT06262139) evaluating MT218's tumor detection capabilities 57.

Here, we demonstrate how MT218-MRMI predicts tumor responses to three immunotherapeutic approaches in an orthotopic, allograft-based murine model of PDAC: a VISTA-blocking mAb 15, 16, a peptide vaccine containing mutant KRAS^G12D^ peptide with adjuvants (R848 and CpG oligonucleotide, TLR 7/8/9 agonists) 5, 58, 59, and a combination of both. These therapies stimulate both innate and adaptive immune responses, leading to increased T-cell infiltration and consequently, enhanced EDB-FN secretion in and around tumors. As outlined in **Figure 1B**, MT218-MRMI technology enables early discrimination between treatment outcomes by detecting these molecular changes as signal alterations. Our findings establish MT218-MRMI as a promising tool for non-invasive, high-resolution, longitudinal characterization of the dynamic TME and validate its utility for combined diagnostic imaging and prognostic assessment in precision cancer immunotherapy.

## Results

### MRMI for monitoring and prediction of tumor responses to anti-VISTA mAb therapy

**Figure 2:**
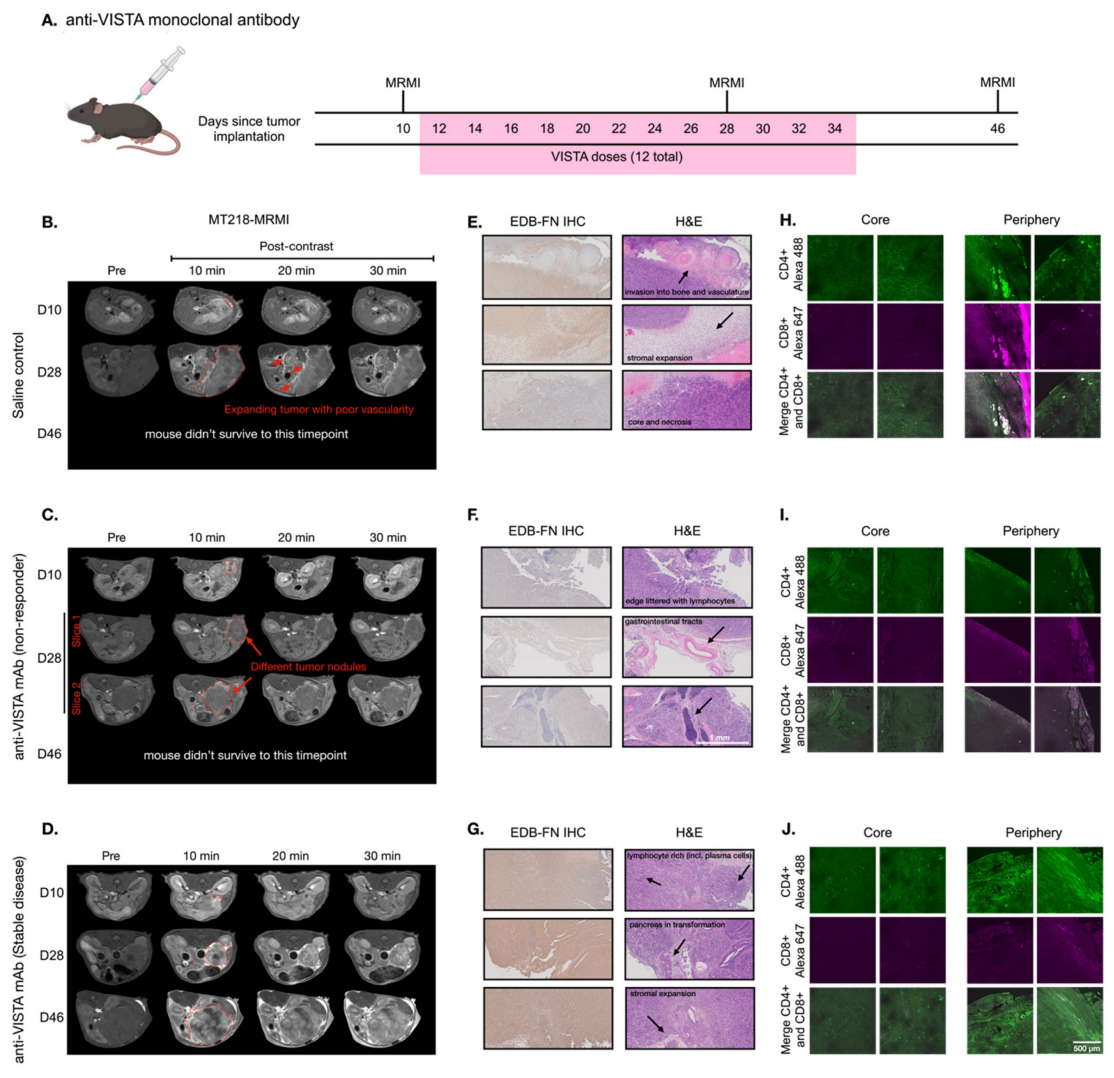
** MT218-MRMI for monitoring tumor response to anti-VISTA mAb in C57BL/6 mice bearing orthotopic KPC-K8484 allografts. A)** Dosing schedule showing anti-VISTA monoclonal antibody treatment and MRMI imaging timepoints (days 10, 28, and 46) after tumor implantation, with 12 total VISTA doses administered between days 12-34. **B-D)** Representative T1-weighted 2D fast spin echo axial MRMI images of mice treated with saline control (**B**) and anti-VISTA mAb (**C**, non-responder; **D**, stable disease) before (pre) and at 10, 20, and 30 minutes after intravenous injection of MT218 (0.1 mmol/kg, post-contrast) on days 10, 28, and 46 after tumor initiation. Tumors are outlined with red dashed lines. **E-G)** IHC of EDB-FN with anti-EDB G4 mAb and H&E staining of tumors from mice treated with saline (**E**) or anti-VISTA mAb (**F**, non-responder; **G**, stable disease) at the end of experiment, showing various tissue characteristics noted by arrows in the H&E panels. **H-J)** IF staining of CD4^+^ and CD8^+^ T cells with anti-CD4^+^ mAb labeled with Alexa 488 (green) and anti-CD8^+^ labeled with Alexa 647 (magenta) in tumors from mice treated with saline (**H**) or anti-VISTA mAb (**I**, non-responder; **J**, stable disease), with separate core and periphery tumor regions displayed.

To investigate how VISTA blockade influences immunotherapy-induced changes in EDB-FN expression, we established an orthotopic PDAC model using KPC-K8484 cells implanted in immunocompetent C57BL/6 mice. Following the experimental schedule shown in **Figure 2A**, mice were treated with a VISTA-blocking monoclonal antibody (mAb). MT218-MRMI confirmed tumor establishment 10 days after cell inoculation, with tumors clearly visible in axial slices adjacent to the spleen (**Figure 2B-D**). Administration of MT218 resulted in an overall signal increase within all tumors in T_1_-weighted MRI, facilitating boundary detection and delineation of tumor masses from the pancreas.

Mice were treated either with saline (100 µL, negative control) or anti-VISTA mAb (200 µg in 100 µL) via intraperitoneal (i.p.) injection three times a week for a total of four weeks starting on day 12. T_1_-weighted MT218-MRMI was performed on days 28 and 46 to monitor tumor response and determine therapeutic efficacy. Non-contrast T_2_-weighted MRI was also used to assess the disease progression of the tumors detected by MT218-MRMI. The mean tumor sizes for various groups as measured by MRI are shown in **Figure S1**. MRMI revealed disease progression in the control group, with lobular tumors exceeding 10% of body weight at endpoint. This aggressive phenotype was characterized by widespread metastasis (liver, kidney, spleen, bone, and muscle) in 63.2% of mice and high mortality (75.9%) by day 46. As shown in **Figure 2B**, day 28 MRMI scans showed heterogeneous enhancement patterns across tumor masses. These findings, supported by tumor growth curves (**Figure S1**), established baseline parameters for assessing treatment response in subsequent therapeutic experiments.

In comparison, anti-VISTA mAb elicited heterogeneous responses among treated mice. Outcomes were characterized by primary tumor size, metastatic tumor presence, and post-contrast signal enhancement pattern. Of 14 treated mice (6 female, 8 male), eight exhibited a non-responder phenotype of large tumors with low contrast enhancement comparable to controls and widespread metastases (**Figure 2C**). Non-responders demonstrated poor survival, with 87.5% not surviving to the third MRMI scan planned for day 46. In contrast, six mice demonstrated stable disease (SD), characterized by smaller tumors compared to controls, no detectable metastases, and distinctly brighter signal enhancement on days 28 and 46 (**Figure 2D**). This group showed markedly improved survival, with 83.3% surviving to day 46.

MT218-MRMI assessment of SD mice showed greater signal enhancement in tumor periphery and moderate tumor core signal on day 28 and 46, indicative of augmented EDB-FN expression within these tumors. Quantitative analysis found that MT218 produced a 3- to 6-fold increase in average normalized contrast-to-noise ratios (CNR) across all tumors compared to pre-treatment (day 10, **Figure S2**). Notably, SD tumors exhibited significantly higher CNR increases (6.17- and 5.75-fold at 10 and 20 minutes post-contrast) on day 28 compared to both controls (3.64- and 3.51-fold) and non-responders (3.44- and 2.82-fold). To validate that enhanced signals resulted from specific binding and not passive diffusion, we compared MT218 to non-targeted gadoteridol in SD tumors after treatment (**Figure S3**) confirming that increased enhancement was due to EDB-FN-specific binding of MT218.

Histological analysis was performed at days 28 and 46 (post-MRMI) or at humane endpoint to characterize treatment responses. IHC using anti-EDB-FN (G4) mAb revealed distinct staining patterns of EDB-FN expression in the tumors across treatment groups: control tumors showed heterogeneous, moderate EDB-FN staining (**Figure 2E**) non-responding tumors displayed relatively weak staining throughout (**Figure 2F**) and the SD tumors exhibited strong staining, particularly intense in the tumor periphery compared to the core (**Figure 2G**). EDB-FN expression patterns, both in intensity and spatial distribution, corroborated MT218-MRMI signal patterns.

Hematoxylin and eosin (H&E) staining revealed distinct histopathological features among treatment groups. Specifically, control and non-responding tumors showed dense adenocarcinoma with minimal pancreatic tissue remnants and little core necrosis **(Figure 2E-F)**. These tumors exhibited aggressive behavior with direct lymphatic invasion and expansion into surrounding tissues including lymph nodes, skeletal muscle, liver, and bones. In contrast, SD tumors had areas of dense adenocarcinoma with substantial core necrosis and retained pancreatic tissue architecture (**Figure 2G**).

T cell infiltration patterns determined tumor immune microenvironment (TIME) phenotypes in response to immunotherapy and were used in tandem with other prognostic factors to predict therapeutic response. Therefore, we performed immunofluorescence (IF) imaging to determine T cell infiltration patterns in response to immunotherapy. Confocal IF revealed that control-treated tumors showed whole-tumor presence of CD4^+^ T cells while cytotoxic CD8^+^ T cells were restricted to the periphery (**Figure 2H**). Non-responding tumors displayed minimal infiltration of both CD4^+^ and CD8^+^ T cells (**Figure 2I**) characteristic of an immune desert phenotype. SD tumors exhibited substantial CD4^+^ T cell presence, particularly at the tumor periphery, with CD8^+^ T cells similarly restricted to peripheral regions (**Figure 2J**) indicating an immune excluded phenotype. The healthy pancreas was also imaged to establish a baseline for normal pancreatic tissue (**Figure S4**).

**Figure 3:**
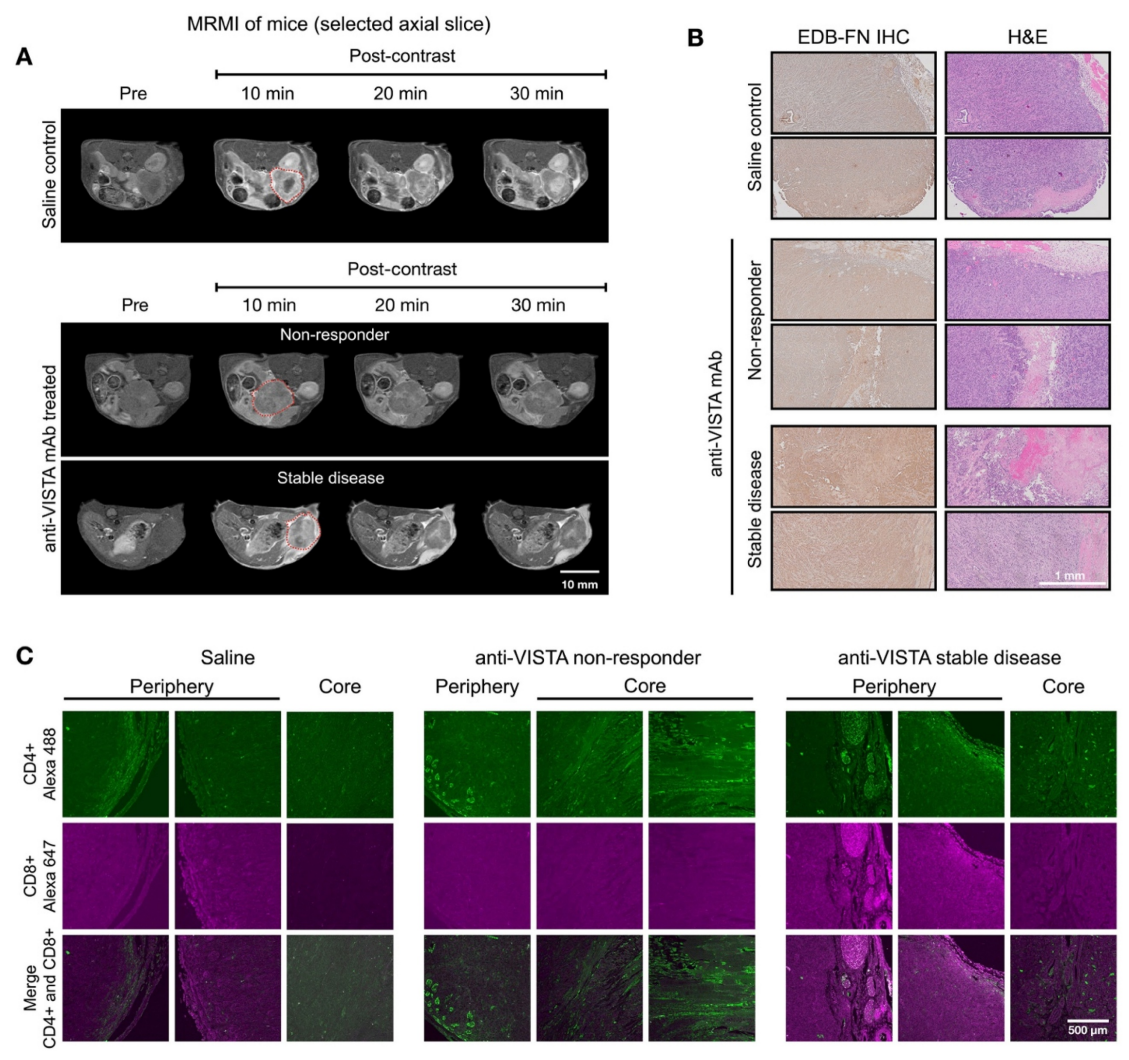
** MT218-MRMI for monitoring early tumor response to anti-VISTA mAb in C57BL/6 mice bearing orthotopic KPC-K8484 allografts. A)** Representative T1-weighted 2D fast spin echo axial MRMI acquisitions of pancreatic tumors (delineated by red dashed lines) from mice administered either saline (control) or anti-VISTA mAb. Images were acquired at baseline (pre-contrast) and at sequential timepoints (10, 20, and 30 minutes) following MT218 administration (0.1 mmol/kg, IV) on day 28 post-implantation, demonstrating differential contrast enhancement patterns corresponding to therapeutic response. **B)** Molecular characterization of the extracellular matrix showing EDB-fibronectin expression via IHC using anti-EDB mAb G4 (left panels) with corresponding H&E staining (right panels) of matched tumor sections. **C)** Immunofluorescence analysis of tumor-infiltrating lymphocytes showing spatial distribution of CD4^+^ T helper cells (visualized with anti-CD4 mAb conjugated to Alexa 488, green) and CD8^+^ cytotoxic T cells (visualized with anti-CD8 mAb conjugated to Alexa 647, magenta), revealing therapy-induced alterations in immune cell recruitment patterns.

We also examined the correlation of MT218-MRMI with the TME phenotypes determined by IF analysis on day 28 after 2 weeks of treatment with saline or anti-VISTA mAb. Response assessment at day 28 revealed that 50% of anti-VISTA mAb-treated mice displayed heterogeneous bright signal enhancement without metastases (**Figure 3A**). SD tumors showed distinctive enhancement patterns, with bright signals in both core and rim regions. Corresponding histological analyses confirmed enhanced EDB-FN expression and reduced cell density in SD tumors compared to non-responders (**Figure 3B**), validating the MT218-MRMI data (**Figure S5**) particularly at the two weeks post-treatment initiation time point.

IF revealed distinct T cell infiltration patterns correlating with treatment response. SD tumors exhibited substantial CD4^+^ and CD8^+^ T cell presence at the tumor periphery, with only CD4^+^ T cells detected in the core (**Figure 3C**). In contrast, non-responding tumors contained sparse CD4^+^ T cells and lacked CD8^+^ T cells that are essential for cytotoxic activity in antitumor immunotherapy 60, 61. The following cellular patterns aligned with MT218-MRMI signal characteristics: SD tumors displayed bright rim enhancement with moderate core signal, reflecting an immune excluded TME, while non-responders showed weak peripheral and core enhancement consistent with an immune desert TME. We determined the correlation between IF staining of CD4^+^ and CD8^+^ markers and MRMI signal measurement in treated tumors (**Figure S6**). Although MRMI could not distinguish CD4^+^ vs CD8^+^ T cells in the tumors, MRMI signal correlated with overall abundance of activated T cells across various treatment groups. Both MRMI and T cell staining showed that anti-VISTA mAb treatment enhanced CD4^+^ T cell infiltration within the tumor core regions in SD hosts. This correlation indicates that MT218-MRMI may reliably distinguish between the different TIME types with distinct therapeutic responses to anti-VISTA treatment.

### MRMI for monitoring and predicting tumor response in response to a cocktail vaccine

**Figure 4:**
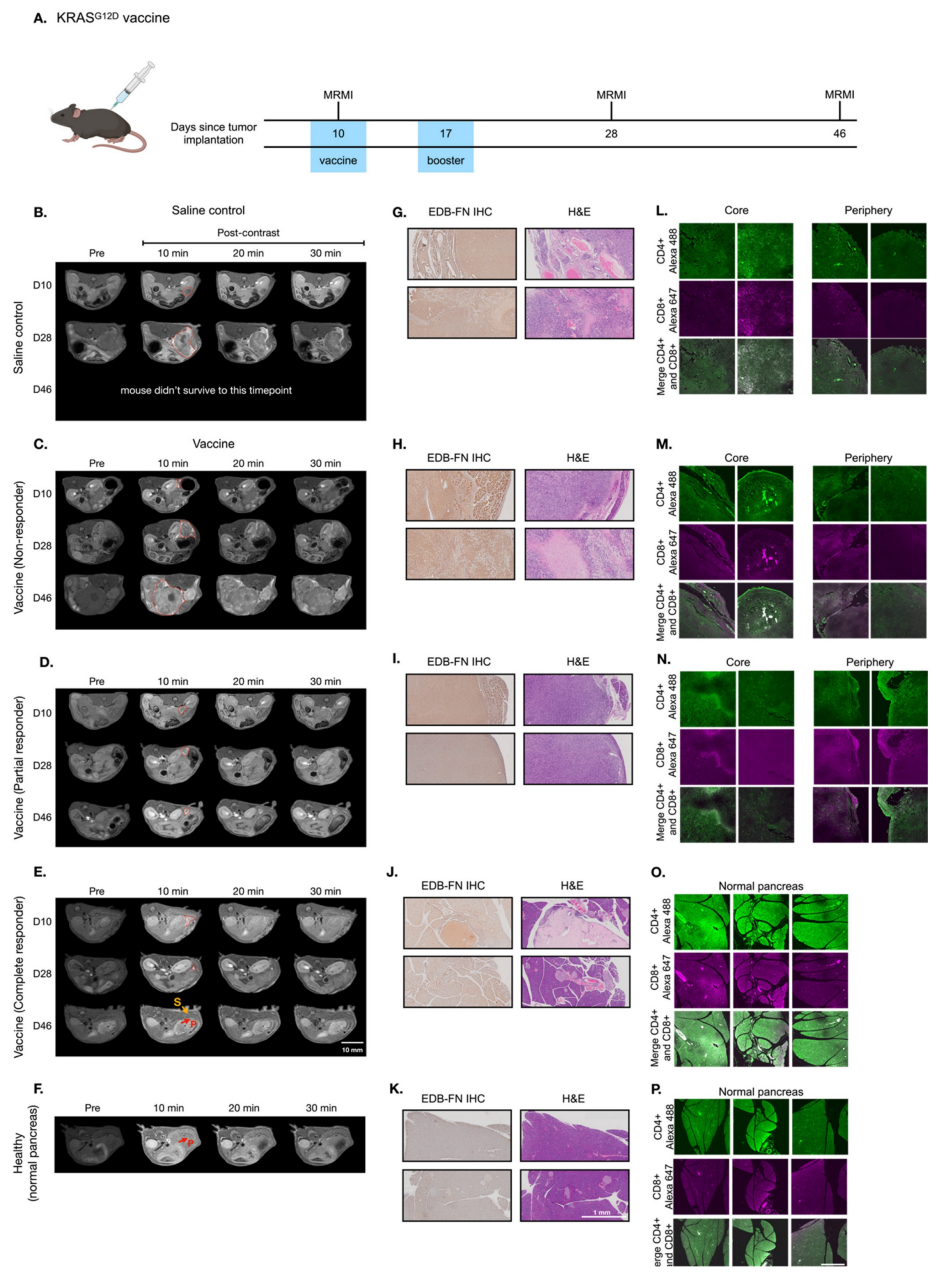
** MT218-MRMI for monitoring tumor response to a mutated KRAS^G12D^ peptide/R848/CpG neoantigen vaccine cocktail in C57BL/6 mice bearing orthotopic KPC-K8484 allografts. A)** Experimental timeline depicting vaccination schedule and longitudinal MRMI acquisition timepoints. **B-E)** Representative T1-weighted 2D fast spin echo axial MRMI images of pancreatic tumors (demarcated by red dashed lines) from saline-treated controls (**B**) and vaccine-treated animals exhibiting varying response patterns (**C-E**). Images were acquired pre-contrast and at sequential timepoints (10, 20, and 30 minutes) following MT218 administration (0.1 mmol/kg, IV), demonstrating differential contrast kinetics. **F)** Corresponding MRMI of healthy pancreata from age-matched control mice showing baseline signal characteristics. **G-K)** Histopathological characterization via immunohistochemical detection of EDB-FN expression with adjacent H&E staining from saline-treated tumor-bearing mice (**G**), vaccine-treated responders with variable response patterns (**H-J**), and healthy pancreatic tissue controls (**K**). **L-P)** Immunofluorescence analysis of T lymphocyte infiltration showing CD4^+^ (Alexa 488, green) and CD8^+^ (Alexa 647, magenta) T cell populations within tumor microenvironment from saline-treated controls (**L**), vaccine-treated animals with different response profiles (**M-O**), and normal pancreatic tissue (**P**), revealing therapy-induced immunomodulation.

In addition to VISTA inhibitor treatment, we investigated whether MT218-MRMI may predict response to a different immunotherapy using neoantigen vaccine. Mice bearing orthotopic KPC tumors were treated with a cocktail of KRAS^G12D^-derived peptide mixed with TLR agonists (R848 and CpG oligonucleotide). Following initial vaccination on day 10 and a booster on day 17 (**Figure 4A**), tumor growth was significantly inhibited (**Figure S7**). MT218-MRMI identified three distinct response patterns in treated tumors: non-responders (3/25 mice), which exhibited tumor growth comparable to the control group with moderate tumor signal enhancement and metastases (**Figure 4C**); partial responders (12/25 mice), which showed reduced tumor growth with bright peripheral enhancement and weak core enhancement (**Figure 4D**); and complete responders (10/25 mice), which displayed bright signals throughout the tumors on day 28 and achieved complete tumor rejection by day 46 (**Figure 4E-F**).

Quantitative analysis showed significantly elevated CNR in complete responders by day 28 (6.46-fold vs pre-treatment 3.57-fold, **Figure S8**), while partial responders showed reduced enhancement (1.80-fold) and non-responders did not show significant change (3.64-fold vs 3.88-fold) at 10 minutes post-injection of MT218. Notably, day 28 MRMI signal patterns strongly predicted treatment outcomes, achieving 90% accuracy in identifying complete response and 85% accuracy in distinguishing non-response from partial response. This predictive capability was validated through survival outcome correlation, with complete responders showing significantly improved survival (median survival > 90 days) compared to partial responders (median survival 62 days) and non-responders (median survival 38 days, p < 0.001).

Histological analysis of vaccine-treated tissues at day 46 validated MRMI findings across response groups (**Figure 4H-J**). Non-responders showed large tumors with metastases, partial responders exhibited smaller tumors without metastases, and complete responders showed no detectable tumors. EDB-FN staining intensity correlated with MRMI enhancement patterns, with stronger staining in non-responder and partial responder tumors compared to complete responder pancreatic tissue. Complete responders maintained normal pancreatic architecture but showed uniform EDB-FN staining in sclerotic nodules, potentially indicating scarring or DAMP-mediated responses after tumor elimination (**Figure 4K**) 62. IF revealed an immune excluded TME in non-responding and partially responding tumors, with CD4^+^ and CD8^+^ T cells concentrated at the periphery (**Figure 4M-N**). Complete responders showed robust CD4^+^ and CD8^+^ T cell infiltration throughout pancreatic tissues.

**Figure 5:**
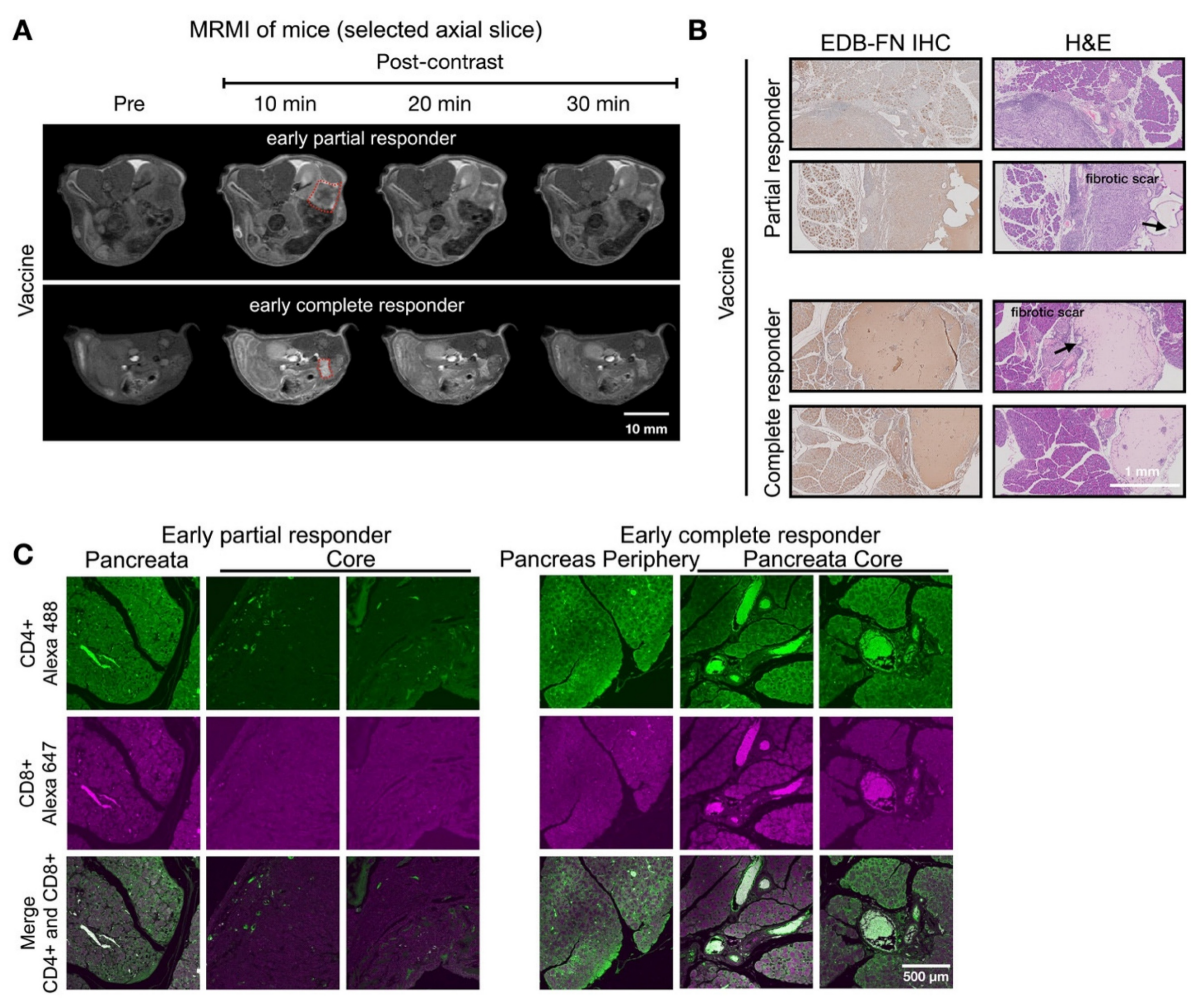
** MT218-MRMI for monitoring early tumor response to a mutated KRAS^G12D^ peptide/R848/CpG neoantigen vaccine cocktail in C57BL/6 mice bearing orthotopic KPC-K8484 allografts. A)** Representative T1-weighted 2D fast spin echo axial MRMI images of tumors (delineated by red dashed lines) from vaccine-treated mice acquired on day 28 post-implantation. Subtraction images demonstrate the differential enhancement pattern between pre-contrast baseline and 10-minute post-MT218 administration, revealing spatial heterogeneity of contrast uptake. **B)** Histopathological analysis showing EDB-FN expression via immunohistochemistry with corresponding H&E staining of tumor sections harvested at experimental endpoint. **C)** Immunofluorescence characterization of intratumoral lymphocyte populations showing spatial distribution of CD4^+^ T helper cells and CD8^+^ cytotoxic T cells within the tumor microenvironment following vaccine therapy.

Response assessment on day 28 demonstrated MT218-MRMI's predictive capability. Two distinct enhancement patterns emerged (**Figure 5A**): larger tumors with strong rim and weak core enhancement (characteristic of partial responders) and smaller tumors with uniform strong enhancement (typical of complete responders). Histologically, responding tumors exhibited scar-like tissue architecture with significant EDB-FN staining while partial responders showed weaker EDB-FN expression (**Figure 5B**). IF revealed minimal CD4^+^ cells at the tumor periphery and sparse CD8^+^ cells in partial responders, in contrast to strong CD4^+^ staining in scar-like tissues and substantial CD4^+^/CD8^+^ T cell accumulation throughout pancreatic tissues in responders (**Figure 5C**). These findings demonstrate MT218-MRMI's ability to detect distinct enhancement patterns within two weeks of treatment initiation, enabling early differentiation of response patterns. Longitudinal assessment further refined response classification based on enhancement patterns, tumor progression, and therapeutic outcomes.

### MT218-MRMI for monitoring and predicting tumor responses to combined treatments with vaccine and VISTA blockade

**Figure 6:**
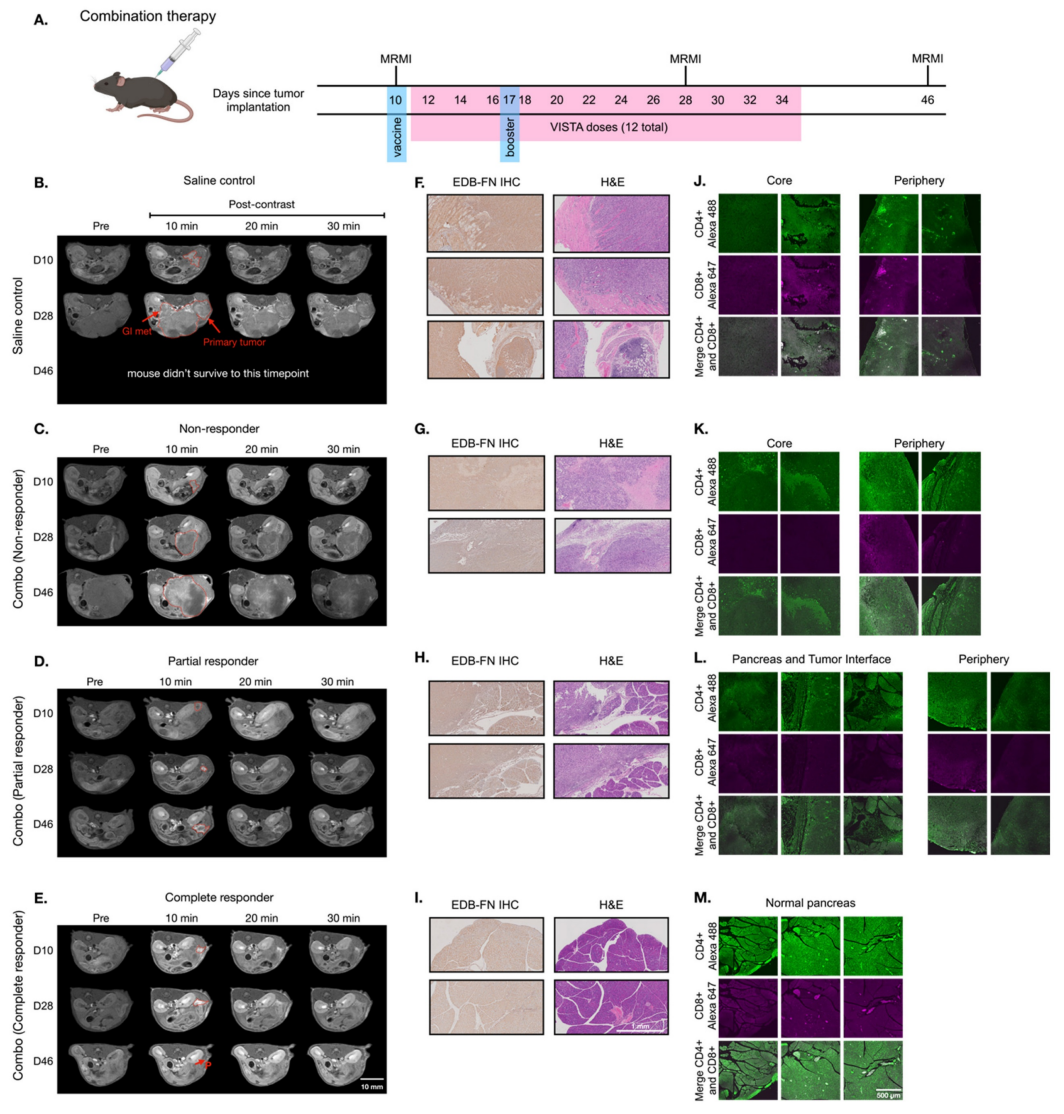
** MT218-MRMI for monitoring tumor response to the combination therapy of the G12D peptide/R848/CpG neoantigen vaccine cocktail and anti-VISTA mAb in C57BL/6 mice bearing orthotopic KPC-K8484 allografts. A)** Treatment schedule showing sequential administration of vaccine and anti-VISTA mAb, with initial vaccine dosing on days 10 and 17. **B-E)** Representative T1-weighted 2D fast spin echo axial MRMI images of pancreatic tumors (demarcated by red dashed lines) from mice receiving saline control (**B**) or combination therapy (**C-E**), acquired pre-contrast and at specified time points post-MT218 administration (0.1 mmol/kg, IV). Images depict differential contrast enhancement patterns correlating with treatment response. **F-I)** Histopathological analysis of tumor sections showing EDB-FN expression via immunohistochemistry with corresponding H&E staining from saline-treated controls (**F**) and combination therapy-treated animals (**G-I**). **J-L)** Immunofluorescence analysis of intratumoral T-cell infiltration showing CD4^+^ (Alexa 488, green) and CD8^+^ (Alexa 647, magenta) T lymphocytes in tissues from saline-treated controls (**J**) versus combination therapy-treated animals (**K-L**), demonstrating therapy-induced immune cell recruitment.

Building on monotherapy findings, we next investigated MT218-MRMI's predictive capability in combination therapy using the vaccine cocktail with anti-VISTA mAb. Following the procedure outlined in **Figure 6A**, PDAC-bearing mice received the initial vaccine on day 10 and a booster on day 17 alongside anti-VISTA mAb treatment (12 doses over 4 weeks, starting on day 12). MT218-MRMI revealed three distinct response patterns (**Figure 6B-E, Figure S9**). Non-responders (4 mice) developed large tumors with weak enhancement (2.74-fold CNR increase) on day 28, progressing to substantial growth with heterogeneous enhancement (3.80-fold CNR increase) and detectable metastases by day 46 (**Figure 6C, Figure S10**). Partial responders displayed reduced tumor volume (< 30% compared to the control growth curve) with bright peripheral enhancement (4.51-fold CNR increase) on day 28. This was followed by tumor regrowth, characterized by distinctive rim signal enhancement and weak core enhancement (2.93-fold CNR) by day 46 (**Figure 6D, Figure S10**). Complete responders (13 mice) exhibited intense signal enhancement (6.94-fold CNR increase) at 10 minutes post-contrast on day 28 and achieved complete tumor resolution by day 46 (**Figure 6E, Figure S10**).

Following the final MRMR scan on day 45, mice were euthanized, and tumor and pancreatic tissue was harvested. IHC, H&E, and IF tissue analysis validated MT218-MRMI findings (**Figure 6F-M**). Complete responders had normal pancreatic tissue structure as compared to complete responders treated with vaccine alone (**Figure 6I**). Non-responders and partial responders showed an immune excluded TME, characterized by substantial whole-tumor CD4^+^ T cell presence and limited CD8^+^ T cells at the tumor periphery with little to no inner tumor presence (**Figure 6J-K**). In contrast, complete responders exhibited robust infiltration of both CD4^+^ and CD8^+^ T cells throughout pancreatic tissue (**Figure 6M**), confirming successful immune activation.

**Figure 7:**
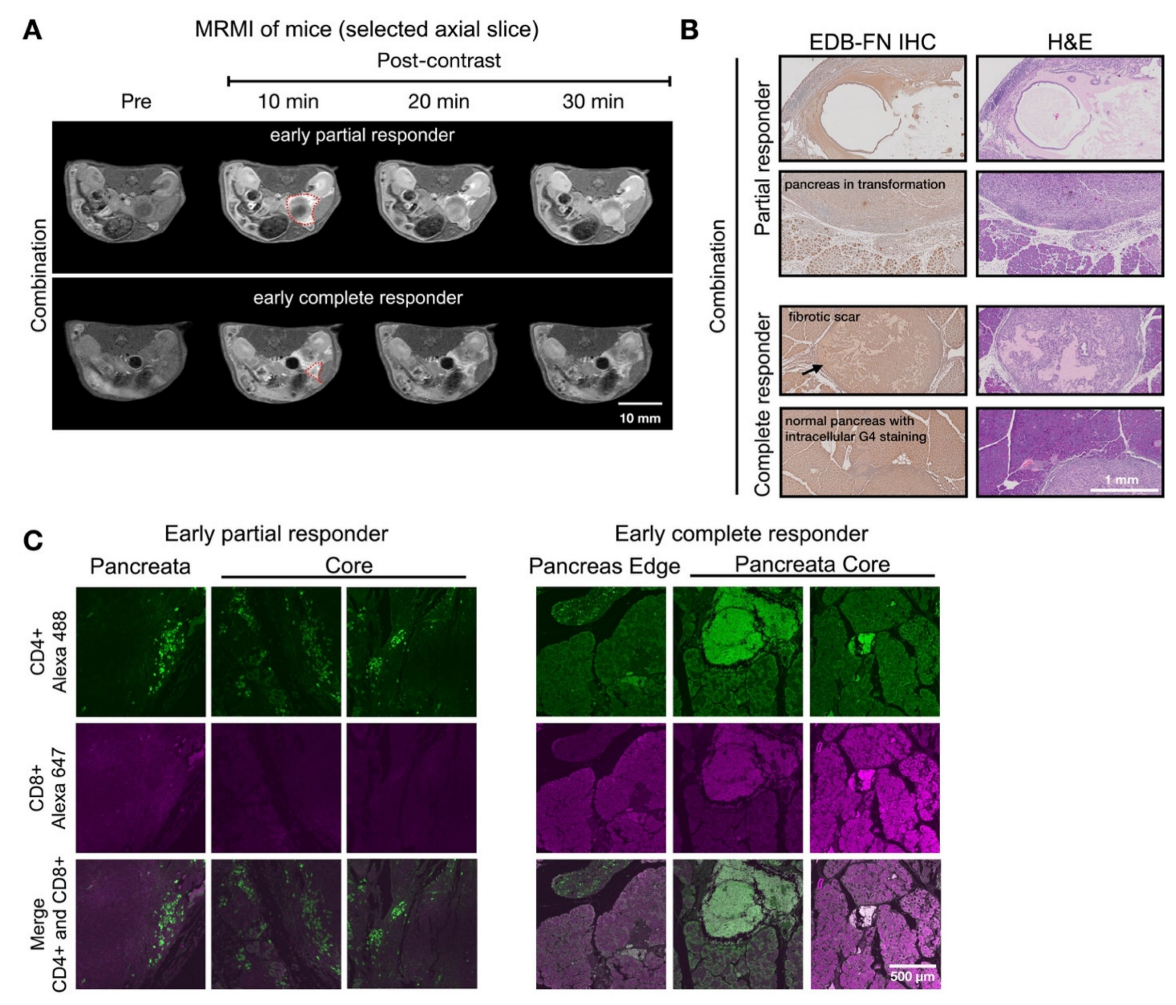
** MT218-MRMI for monitoring early tumor response to anti-VISTA and mutated KRAS^G12D^ peptide/R848/CpG neoantigen vaccine cocktail in C57BL/6 mice bearing orthotopic KPC-K8484 allografts. A)** Representative T1-weighted 2D fast spin echo axial MRMI of tumors (outlined with red dashed lines) from mice treated with combination therapy, acquired on day 28 post tumor implantation. **B)** Immunohistochemical (IHC) analysis of EDB-FN expression and H&E staining of tumor sections harvested at experimental endpoint. **C)** Immunofluorescence (IF) staining of CD4^+^ T cells (green) and CD8^+^ T cells (magenta) in tumor sections demonstrating immune cell infiltration patterns following combination therapy.

**Figure 7** showed two distinct MT218-MRMI signal enhancement patterns on day 28 post-treatment: strong rim enhancement with weaker core enhancement in large tumors (early partial responders) and uniform strong enhancement across smaller tumors (early responders) at 10 minutes post-injection. These signal patterns are similar as those in vaccine-treated tumors, (**Figure 5**). IHC analysis confirmed that stronger MRMI signal enhancement correlated with higher EDB-FN expression (**Figure 7B**). Unlike vaccine-treated tumors, early responders showed no scar-like tissues (**Figure 7**). IF analysis demonstrated CD4^+^ and CD8^+^ T cell infiltration at tumor peripheries and pancreas-adenocarcinoma borders in partial responders (**Figure 7C**). Early responders exhibited robust CD4^+^ and CD8^+^ T cell presence throughout adenocarcinoma regions and adjacent pancreatic tissue. These findings demonstrate MT218-MRMI's capability to detect early immunotherapy responses through signal enhancement patterns and tumor size changes, making it an effective tool for monitoring tumor progression and treatment outcomes.

### Predicting long-term therapeutic outcomes with MRMI

Using early-stage MT218-MRMI signal data, we developed a Cox proportional hazards model to classify therapeutic outcomes in PDAC-bearing mice. Imaging analysis identified distinct response patterns differing in signal enhancement and immune characteristics. Non-responders exhibited low MRMI signal enhancement throughout tumors, corresponding to an immune desert TME, rapid tumor growth, and metastasis. SD cases showed bright tumor periphery with moderate core enhancement, reflecting an immune excluded TME and rapid tumor growth without metastasis. Partial responders displayed bright tumor periphery with weaker core enhancement, also indicating an immune excluded TME but with inhibited tumor growth and no metastasis. Finally, complete responders demonstrated uniform bright enhancement throughout the tumor, correlating with an immune inflamed TME and no detectable tumor or metastasis.

To validate this classification model, we analyzed a larger cohort of PDAC-bearing mice treated with saline (control n = 20-29, dependent on group), anti-VISTA mAb (n = 14), vaccine (n = 27), or combination (n = 28) therapy, evaluating MT218-MRMI's ability to detect early changes at 2 weeks post-treatment and predict long-term outcomes. The model achieved 82.9% overall accuracy in predicting tumor growth and survival outcomes, with treatment-specific accuracies of 100% for saline control, 76.9% for vaccine treatment, and 71.4% for combination therapy. Notably, the model showed perfect prediction accuracy for both complete responders and non-responders (100%), with reduced accuracy for partial responders (22.2%).

**Figure 8:**
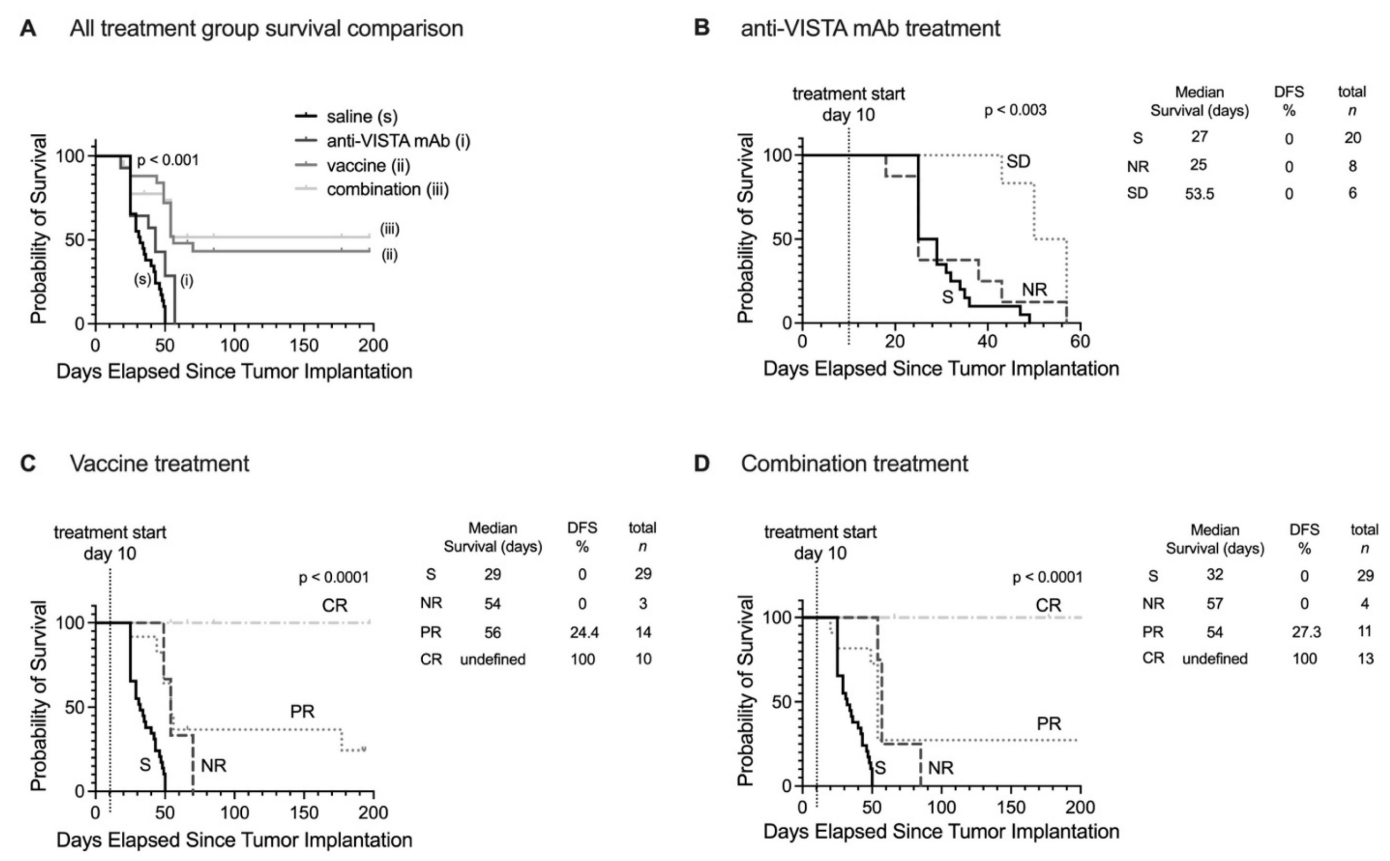
** Prediction of therapeutic outcomes based on MT218-MRMI response criteria. A**) Kaplan-Meier survival curves comparing overall survival across all PDAC treatment groups (saline control, anti-VISTA mAb, vaccine cocktail, and combination therapy). **B-D)** Stratified survival analyses based on MT218-MRMI response classification showing distinct prognostic groups: non-responders (NR), stable disease (SD), partial responders (PR), and complete responders (CR) within the anti-VISTA mAb group (**B**), the KRAS vaccine cocktail group (**C**), and the anti-VISTA plus vaccine combination therapy group (**D**), all compared against the saline control group (S).

Survival analysis extending to 200 days post-treatment revealed distinct patterns across treatment groups (**Figure 8**). Saline and anti-VISTA mAb groups showed no tumor-free survival (**Figure 8A**), while vaccine treatment achieved 43.2% overall disease-free/tumor-free survival and combination therapy improved survival to 51.6%. When stratified by MT218-MRMI response, no tumor-free survival was observed in non-responders or stable disease groups across all treatments (**Figure 8B-D**). Partial responders achieved 24.2% disease-free survival with vaccine therapy (**Figure 8C**) and 27.3% with combination therapy (**Figure 8D**). Most significantly, complete responders in both vaccine and combination therapy groups maintained 100% tumor-free survival throughout the 200-day monitoring period, validating the predictive value of early MT218-MRMI signal patterns.

These results demonstrate that MT218-MRMI effectively monitors tumor immunotherapy response through distinct enhancement patterns correlating with immune activation, TME characteristics, and long-term survival outcomes. The high overall accuracy of the classification model, 82.9%, particularly in identifying complete responders and non-responders, supports MT218-MRMI's potential as a predictive imaging tool for monitoring and predicting immunotherapy outcomes.

## Discussion

Despite significant advances, existing clinical imaging tools remain limited in assessment of immunotherapy response, particularly for pancreatic cancer. Enabling high resolution visualization of soft tissue with excellent sensitivity for pancreatic and PDAC tissue, MRMI is an ideal modality for accurate and non-invasive characterization of immunotherapy response 63. Our previous studies demonstrated that MRMI of EDB-FN with MT218 is effective in detecting multiple aggressive cancers, including PDAC, and in monitoring tumor response to anticancer therapies in preclinical models 33, 48, 56, based on the role of EDB-FN in cancer EMT and invasion. This study further shows that MT218-MRMI of EDB-FN is effective in monitoring tumor responses to immunotherapy and predicting therapeutic outcomes in a preclinical PDAC model, leveraging its biological function as a T cell activator. Cellular FN, including EDB-FN, functions as an ECM protein that facilitates cell adhesion and migration. Both aggressive cancer cells and activated T cells secrete high levels of EDB-FN to promote invasion or migration, not by normal cells, providing a dynamic stromal marker which can be targeted by high-resolution MRMI for accurate detection of aggressive tumors as well as monitoring and prediction of treatment response 33, 41, 45.

MRMI offers several unique advantages for high-resolution and dynamic characterization of TME changes at any tissue depth, making it particularly valuable for visualizing pancreatic tumors often obscured by surrounding organs. MT218-MRMI in particular revealed heterogeneous contrast enhancement patterns that reflect variable EDB-FN expression levels and distribution throughout immunotherapy treatment. MT218 generated strong signal enhancement 10-20 minutes post-injection, which gradually diminished over time. This gradual decline in signal intensity can be attributed to MT218's moderate binding affinity (K_a_ = 3.5 µM), diffusion, and clearance 51. However, no clear correlation between diffusion patterns and therapeutic outcomes was established as several factors influence the agent's diffusion. For instance, intratumoral degradation of MT218's targeting peptide, ZD2, by ECM peptidases could accelerate its clearance. Tumor necrosis could further affect contrast agent diffusion, leading to dynamic changes in enhancement patterns. Therefore, we focused on MRMI imaging 10 minutes post-injection in order to obtain the most accurate representation of MT218 binding to EDB-FN for assessing tumor responses to immunotherapies.

Immunotherapy efficacy depends on helper CD4^+^ and cytotoxic CD8^+^ T cells, with T cell distribution strongly correlating with both imaging patterns and survival outcomes (Spearman's ρ = 0.83, p < 0.001). Previous studies have established the multifunctional role of oncofetal FN subtypes in T lymphocyte development, activation, and infiltration, with activated CD4^+^ and CD8^+^ T cells expressing and secreting high levels of EDB-FN 31, 36-40. Wagner *et al.* further showed that anti-CD3 activated CD4^+^ and CD8^+^ T cells expressed and resulted in high levels of EDB-FN exertion 45. Our spatial and temporal analyses revealed distinct T cell distribution patterns corresponding to therapeutic response: non-responders showed characteristics of immune desert (limited T cell presence, mean density < 50 cells/mm²), partial responders demonstrated immune exclusion phenotype (peripheral T cell accumulation, 245 ± 35 cells/mm² peripherally vs 42 ± 12 cells/mm² centrally), and complete responders exhibited an inflamed immune phenotype (abundant intratumoral T cells, mean density 325 ± 45 cells/mm² throughout).

**Figure 9:**
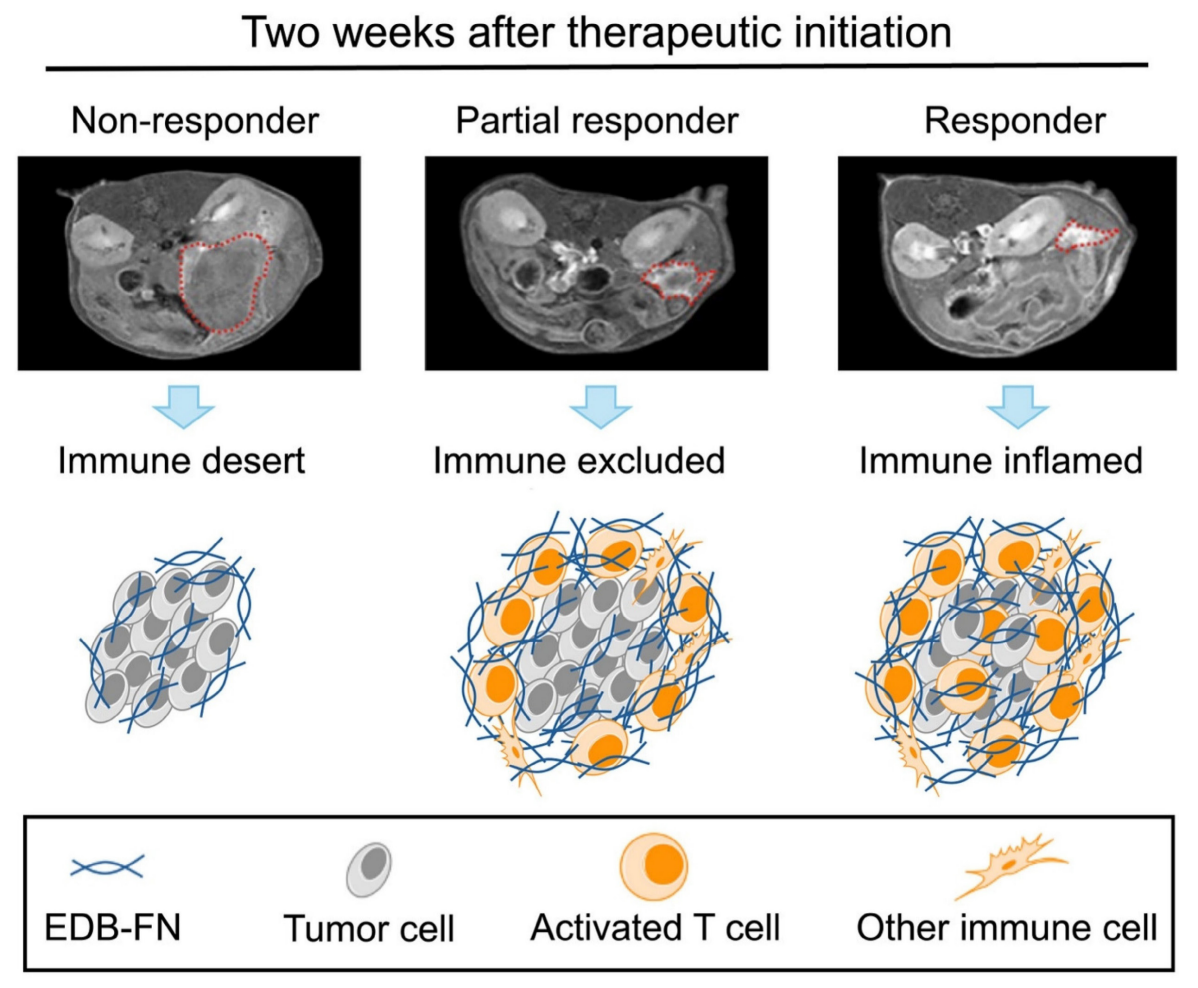
** MT218-MRMI tumor signal enhancement patterns correlate with distinct immune tumor microenvironment (TME) phenotypes in immunotherapy response groups.** MT218-MRMI shows heterogeneous signal enhancement patterns within tumors (indicated by red dashed lines) and imaging can be used to comprehensively predicts immunotherapy response in pancreatic cancer. MT218-MRMI derived signal enhancement patterns reflect characteristic differences in EDB-FN expression that correspond to three distinct TME phenotypes: immune desert, immune excluded, and immune inflamed.

Analysis at day 28 showed responding tumors with significant CD4^+^ and CD8^+^ infiltration (> 200 cells/mm²) either peripherally or throughout the tumor mass, correlating with MRMI enhancement patterns. By day 46, extensive CD4^+^ presence but limited CD8^+^ infiltration was seen in stable/partial responders. While specific CD4^+^ subtypes (Foxp3^+^ Tregs vs Foxp3^-^ helper cells) were uncharacterized, enhanced MT218-MRMI signals likely reflect combined tumor and T cell EDB-FN secretion, providing a dynamic TME marker of therapeutic response. These imaging patterns corresponded with treatment outcomes, as demonstrated by Cox proportional hazards analysis showing significant survival benefits for both vaccine (HR = 0.43, 95% CI: 0.25-0.74, p = 0.002) and combination therapy (HR = 0.38, 95% CI: 0.22-0.66, p = 0.001) compared to saline controls. We hypothesize that the increased tumor signal enhancement in MT218-MRMI following immunotherapy may be associated with additional EDB-FN secretion by activated CD4^+^ and CD8^+^ T cells. Taken together, these results support the conclusion that the enhancement patterns detected by MT218-MRMI during immunotherapy reflect distinct types of tumor microenvironment in response to treatment (**Figure 9**).

Significant efforts are focused on developing molecular imaging tools to specifically target immune cell surface markers. Several PET probes have been engineered to visualize CD69 64, CD8 28, 65, fibroblast activation protein (FAP) 66, and immune checkpoints 67. Despite PET's sensitivity for molecular imaging in both preclinical and clinical investigations, its spatial resolution limitations (4-5 mm) preclude detailed visualization of TME phenotypic alterations following immunotherapeutic intervention. A case in point: while PET imaging using 89Zr-labeled one-armed antibody (89Zr-ED88082A) successfully tracked CD8^+^ T cell populations, it failed to provide definitive assessment of tumor responsiveness to immune checkpoint blockade in patients with colorectal cancer liver metastases 28. Similarly, ^68^Ga-FAPI PET/CT effectively monitored dynamic changes in cancer-associated fibroblasts (CAFs) and demonstrated superior performance to ^18^F-FDG PET in evaluating tumor response to immune checkpoint inhibition in metastatic colorectal cancer patients 66. MRMI, however, offers distinct advantages over PET, including superior three-dimensional and enhanced soft tissue visualization with high spatial resolution. Our data demonstrates that MT218-MRMI generates high-resolution images with characteristic contrast enhancement patterns reflective of EDB-FN expression in tumors that correlate with immunotherapeutic response profiles. These distinct imaging signatures effectively capture dynamic TME remodeling that reflect the dynamic infiltration of activated immune cells and are correlated with responses to immunotherapies.

Therapeutically, we have also demonstrated that anti-VISTA mAb monotherapy prevented metastatic spread in KPC tumors and extended survival in stable disease, with significant improvement in survival probability (log-rank p < 0.001). The KRAS^G12D^ peptide vaccine improved survival (HR = 0.43, 95% CI: 0.25-0.74) and reduced tumor burden, while the combination of vaccine and anti-VISTA treatment achieved optimal tumor-free survival (HR = 0.38, 95% CI: 0.22-0.66). The combination treatment of anti-VISTA mAb and KRAS^G12D^ peptide vaccine demonstrated optimal therapeutic efficacy, preventing metastatic spread while achieving tumor-free survival in complete responders. Partial responders achieved a median survival of 54-56 days compared to 27-32 days in non-responders, whereas complete responders did not reach median survival during the monitoring period. These results both establish the utility of MT218-MRMI for monitoring immunotherapy response and demonstrate the therapeutic potential of combining checkpoint blockade with targeted vaccination for the treatment of KRAS^G12D^-mutated pancreatic cancer.

This investigation has several limitations. MRMI images and IHC/IF stains were not perfectly co-registered due to natural variation in the tissue handling process which resulted in tissue shrinkage and anatomical misalignment. These processing methods made it difficult to precisely correlate the MRMI data with activated T cells in the tumors. Future studies should consider the use of custom rigs in the MR bed and novel fixing methods to allow for improved co-registration. In addition, MRMI could not distinguish between activated CD4^+^ and CD8^+^ T cells because both secrete EDB-FN. Future studies will investigate the mechanisms that induce the secretion of EDB-FN in T cells. Moreover, we plan to further validate the effectiveness of MT218-MRMI with established immunotherapies, particularly vaccine adjuvants in combination with clinically approved immune checkpoint inhibitors (ICIs), across multiple cancer types. Concurrent optimization of imaging intervals will be critical for maximizing early response prediction capabilities. Additional validation studies in larger cohorts encompassing diverse PDAC subtypes will facilitate the broader clinical application of the MT218-MRMI approach.

### Outlook

MT218-MRMI is capable of monitoring immunotherapy responses through semi-quantitative imaging, as demonstrated here in PDAC. As an FDA-approved agent for cancer detection currently under clinical investigation 57, MT218-MRMI enables high-resolution, non-invasive molecular imaging of the tumor microenvironment (TME) and capturing of treatment response heterogeneity. MT218-MRMI could provide early characterization of treatment responses to classify non-responders, individuals with stable disease, partial responders, and complete responders: this stratification would be invaluable for informing precision immunotherapy. Given the lack of reliable predictive imaging tools for PDAC patients receiving immunotherapy, MT218-MRMI represents a significant advancement in image-guided treatment monitoring. Our findings establish MT218-MRMI as a promising platform for monitoring immunotherapy response in PDAC which warrants further clinical investigation and development. Successful clinical translation of MT218 has the potential to provide real-time, non-invasive assessment of therapeutic response, transform clinical decision-making, and ultimately improve patient outcomes.

## Methods

### Cell culture

Murine Kras^G12D/+^; P53^R172H/+^; Pdx1-Cre PDAC cells (KPC-K8484 or KPC), derived from an inducible murine model, were provided by Dr. Jordan Winter (University Hospitals Cleveland Medical Center, Cleveland, OH). Cells were transfected with lentivirus to express GFP and Luciferin. Cells were cultured in an incubator at 37 °C and 5% CO_2_ with normal RPMI media (RPMI medium (Gibco, Waltham, MA) supplemented with 10% fetal bovine serum (Gibco, Waltham, MA) and 1% Penicillin/Streptomycin (Thermo Fisher Scientific)). Cells were split twice a week for a maximum of 12 passages to prevent significant genetic changes.

### Animals care and tumor model establishment

Seven-week-old male and female C57BL/6 mice (The Jackson Laboratory, Bar Harbor, ME) were used for *in vivo* experimentation. Mice were housed in the Case Western Reserve University Small Animal Imaging Center, in accordance with IACUC approved animal manipulation protocols. To recapitulate the tumor microenvironment, therapy distribution conditions and contrast uptake preclinical tumors were implanted into the pancreas. KPC-GFP-Luc cells were implanted in mice via pancreatic laparotomy while mice were under anesthesia with 2% isofluorane (Covetrus, Partland, ME) and O_2_ (1L/min). Orthotopic tumors were established whereby 5000 KPC cells suspended in a 30 mL PBS-Matrigel mixture, at a 2:1 ratio, and were injected into the pancreas. Following a 10-day surgical recovery period, staples were removed and mice were imaged in a 3.0T small animal MRI scanner (MRS*DRYMAG, MR Solutions, Surrey UK), to establish a baseline prior to treatment. Treatment was initiated when tumors were clearly visualized and had reached sizes of > 20 mm^3^. Volumes were computed by ROI measurement over slice areas and summed, gaps of 0.2 mm between slices were accounted for in volume calculations. To validate implantation prior to MRMI, tumor growth was also monitored using bioluminescence imaging on a IVIS Spectrum (Perkin Elmer, Waltham, MA, USA). For preliminary and validation studies, mice were implanted with subcutaneous flank tumors with 100,000 KPC-GFP-Luc in a 1:1 PBS-Matrigel mixture. 100 mL was injected into the flank and tumors were allowed to grow until they had reach 100 mm^3^ before imaging.

### Immunotherapy injections and formulation

Five distinct groups of mice were utilized for this study: negative control (no surgery), positive control (surgery, i.p. saline dosed as a imaging control based on past precedent), anti-VISTA mAb-treated (ICI therapy, clone #13F3, BioXCell Inc, Lebanon, NH), peptide vaccine cocktail of (KRAS-G12D peptide (KLVVVGADGVGKSALTI) 5 (Atlantic Peptides, Concord, NH) and TLR7/8/9 agonists, resiquimod (R848) and CpG-ODN Class B 1826 (InvivoGen, San Diego, CA), and the combination of anti-VISTA mAb and peptide vaccine cocktail. Intraperitoneal (i.p.) injections of 200 mg anti-VISTA mAb were given to mice 3 times a week, at most every other day, to relevant groups beginning on day 12 after tumor initiation. The vaccine cocktail was administered at a dose of 25 mg R848, 20 mg G12D peptide, and 15 mg CpG-ODN on day 10 (following MRMI, prior to ICI therapy) and 17 after tumor initiation. Combination treatments were performed by i.p. injection of anti-VISTA mAb and the vaccine cocktail with same doses and schedule as each individual treatment. Mice in the control group received equivalent volumes (saline) at the same dosing schedule as the relevant treatment group.

### Image acquisition

A 3.0T small animal MRI scanner (MRS*DRYMAG, MR Solutions, Surrey, UK) with a short mouse coil was used to acquire all images. Images were acquired at four time points: prior to contrast injection (pre), 10 minutes post-contrast injection (10 min), 20 minutes post-contrast injection (20 min), and 30 minutes post-contrast injection (30 min). At each time point, a T_1_-weighted axial fast spin echo scan (T_1_w FSE; TE: 11 ms, TR: 305 ms, slice thickness: 1 mm, matrix: 256 x 256, 4 averages, flip angle: 90) and a 3D fast low angle shot (3D FLASH; TE: 3.8 ms, TR: 23 ms, slice thickness: 0.2 mm, matrix: 128 × 256 × 128, 2 averages, flip angle: 25°) scan based on reconstruction of coronal slices was acquired. Additionally, a non-contrast T_2_-weighted coronal FSE (TE: 68 ms, TR: 5000 ms, averages 1, slice thickness 1 mm) scan was acquired to measure tumor size and fluid content of the tumor core, the peritumoral region, and the extratumoral space.

### Image analysis

Region-of-interest (ROI) image analysis was performed using FIJI open-source software (https://imagej.net/contribute/citing) and custom MATLAB software. Pancreatic tumors were localized using the kidney and spleen as anatomical landmarks. Conventional contrast-to-noise ratio (CNR) analysis involved drawing ROIs of whole tumors, one muscle region that served as control normal tissue, and background for noise. ROI analysis was performed for every T_1_-weighted axial slice containing tumor. CNR was calculated using the following: 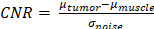. CNR values were calculated for MRI acquired for each time point and post-contrast CNR values were normalized to pre-contrast CNR for comparison. To mitigate bias, CNR calculations were performed independently by two individuals (one blinded). Images were adjusted to the same window and level based on muscle tissue for image subtraction. Volume segmentation was conducted using ITK-SNAP (https://www.itksnap.org/pmwiki/pmwiki.php?n=Main.Publications) and FIJI by collecting ROI measurements over the volume of the tumor and summation. Volume measurements were conducted by two individuals (one or both blinded).

For coronal image analysis of T_2_w and FLASH scans, each coronal MRI sequence was interpolated into a 3D scan and processed in Horos (https://horosproject.org/about/) and FIJI. ROIs were drawn around the spleen, liver, tumor, muscle, and background. CNR values were calculated as described above. Three-dimensional scans were sliced along the Z-axis and the slice with mean tumor enhancement was chosen for analysis.

### Histological staining and grading

Following euthanasia, tumors, livers, kidneys, and spleens were dissected and fixed in formalin for 24 hours. Tissues were then stored in 70% ethanol and labeled in cassettes. Histological staining was conducted by the tissue resource core at the Case Cancer Comprehensive Center. Tissues were sectioned at 5 µm and fixed to coverslip slides. Hematoxylin and eosin (H&E) staining was conducted following standard procedure, and immunohistochemistry (IHC) was performed using an anti-G4 mAb (Absolute Antibody, Boston, MA) at a ratio of 1:100. Briefly, after deparaffinization, antigen retrieval was performed at 125 °C for 30 seconds in citrate buffer (pH = 6.0), followed by blocking with 3% H_2_O_2_ peroxidase and Background Sniper (BioCare Medical, Pacheco, CA) for 8 and 20 minutes, respectively. Primary anti-G4 (for EDB-FN) antibody was incubated with tissue sections at RT for 1 hour with agitation. Primary antibody detection was performed with Mach 3 Rabbit detection solution (BioCare Medical) for 30 minutes. Visualization was performed with betazoid DAB (BioCare Medical, Pacheco, CA) for 5 minutes, followed by a 30 second counterstain with hematoxylin (BioCare Medical). Stained slides were imaged with a Bx61WS (Olympus, Waltham, MA) slide scanner and processed in the associated OlyVIA software. Histological interpretation was conducted by a board-certified pathologist blinded to sample groups.

### Immunological assays

Dual immunofluorescence (IF) staining was performed on OCT-embedded tissue sections. Samples were brought to room temperature, washed twice with PBS to remove OCT, and a hydrophobic barrier was drawn around the tissue sections. The tissue was fixed with 100-200 µL of ice-cold acetone per slide, incubated at -20 °C for 5-10 minutes, and washed three times with PBS. Blocking was performed for 30-60 minutes with 5% serum goat (Sigma-Aldrich, St. Louis, MO) in PBS-T (0.1% Tween-20 in PBS). A cocktail of primary antibodies with mouse reactivity, each raised in different species, was prepared in 1% serum in PBS-T. Primary antibodies targeting CD4^+^ and CD8^+^ (rat anti-CD4^+^ and rabbit anti-CD8^+^, Thermo Fisher Scientific, Waltham, MA) were applied to the tissue sections and incubated at room temperature for 1-2 hours, followed by overnight incubation at 4 °C in a humidified chamber. After washing with TBS-T, a cocktail of species-specific secondary antibodies conjugated to distinct fluorophores (Alexa Fluor 488 anti-rat and Alexa Fluor 647 anti-rabbit, Thermo Fisher Scientific) was applied to the sections and incubated in the dark at room temperature for 1-4 hours. Following secondary antibody staining, the slides were washed, mounted with DAPI-containing mounting medium (Thermo Fisher Scientific), and coverslipped. The slides were sealed with nail polish, dried for 5 minutes, and imaged on an Olympus FluoView FV1000 confocal microscope (Olympus, Tokyo, Japan) at 10x magnification. All slides were stored in the dark at 4 °C or -20 °C. Alexa 488 and Alexa 647 were imaged and set to green and magenta, respectively. Maximum signal was determined based on normal spleen sample and intensity was kept consistent amongst samples. Twelve slices were acquired between the range where cells were visible. Z-stacks were reconstructed at maximum intensity in MATLAB based on the acquired tiff files. Histogram analysis was conducted by thresholding CD4 and CD8 stained slides, which were all acquired under the same confocal conditions. The images were thresholded prior to analysis and the ImageJ histogram feature was used to acquire pixel values.

### Classification and survival analysis

A Cox proportional hazards regression model was developed for tumor response classification model using early-stage MT218-MRMI signal patterns. Images were acquired at 10 minutes post-injection, as this time point provided optimal visualization of MT218 binding to EDB-FN. Enhancement patterns were quantified using a semi-automated region-of-interest (ROI) analysis to calculate signal-to-noise ratios and enhancement distribution metrics (peripheral:core ratio, uniformity index). The classification model incorporated both quantitative imaging features and histologically confirmed immune characteristics to categorize responses as non-responder, stable disease, partial response, or complete response. Model performance was evaluated through leave-one-out cross-validation in a cohort of 41 PDAC-bearing mice treated with saline (n = 14), VISTA (n = 10), vaccine (n = 13), or combination therapy (n = 14). Kaplan-Meier survival analysis was performed with outcomes monitored for up to 200 days post-treatment, and statistical significance was assessed using log-rank tests. Hazard ratios (HR) with 95% confidence intervals (CI) were calculated to quantify treatment effects relative to saline controls. Classification accuracy was evaluated globally and by response category, with separate analyses for each treatment group to assess model robustness across different therapeutic interventions.

### Statistical analysis

GraphPad Prism 9 and 10 were used for statistical analyses. Students t-test (two-tailed, unpaired) was employed for categorical variables. Two-way ANOVAs with Tukey's post hoc test was used longitudinal MRMI signal analysis between groups and for comparisons of outcomes between therapeutic groups. Kaplan-Meier analysis was utilized for survival studies. A two-sided p < 0.05 was considered statistically significant.

## Supplementary Material

Supplementary figures.
