# Supplementary material for: MR molecular imaging for monitoring and predicting tumor responses to immunotherapy
Source: Theranostics. 2025 Sep 22;15(18):10045–63. doi: 10.7150/thno.106481 (PMC12486160; doi:10.7150/thno.106481)
Supplement: Supplementary file 1 — Supplementary figures. [file thnov15p10045s1.pdf]

Supplemental Materials

MR molecular imaging for monitoring and predicting tumor responses to immunotherapy in pancreatic cancer

Victoria E. A. Laney[1], Walter Zhao[1,2], Inga M. Hwang[1,2], Emma Hampson[1], Juan Dong[3], Ryan C. Hall[1], Kristin Weber-Bonk[4], Xueer Yuan[1], Elizabeth Delaney[3], Hannah Gilmore[5,6], Ruth Keri[4,6], Jordan Winter[6,7], Li Lily Wang[3,6*] and Zheng-Rong Lu[1,6,*]

[1]Department of Biomedical Engineering, Case Western Reserve University, Cleveland, OH 44106, USA.

[2]Medical Scientist Training Program, Case Western Reserve University School of Medicine, Cleveland, OH 44106, USA.

[3]Department of Translational Hematology and Oncology Research, Lerner Research Institute, Cleveland Clinic Foundation, Cleveland, OH 44106, USA.

[4]Department of Cancer Biology, Lerner Research Institute, Cleveland Clinic Foundation, Cleveland, OH 44195, USA.

[5]Department of Pathology, Cleveland Clinic Foundation, Cleveland, OH, 44195, USA.

[6]Case Comprehensive Cancer Center, Case Western Reserve University, Cleveland, OH 44106, USA.

[7]Department of Surgery, University Hospitals Cleveland Medical Center, Cleveland, OH 44106, USA.

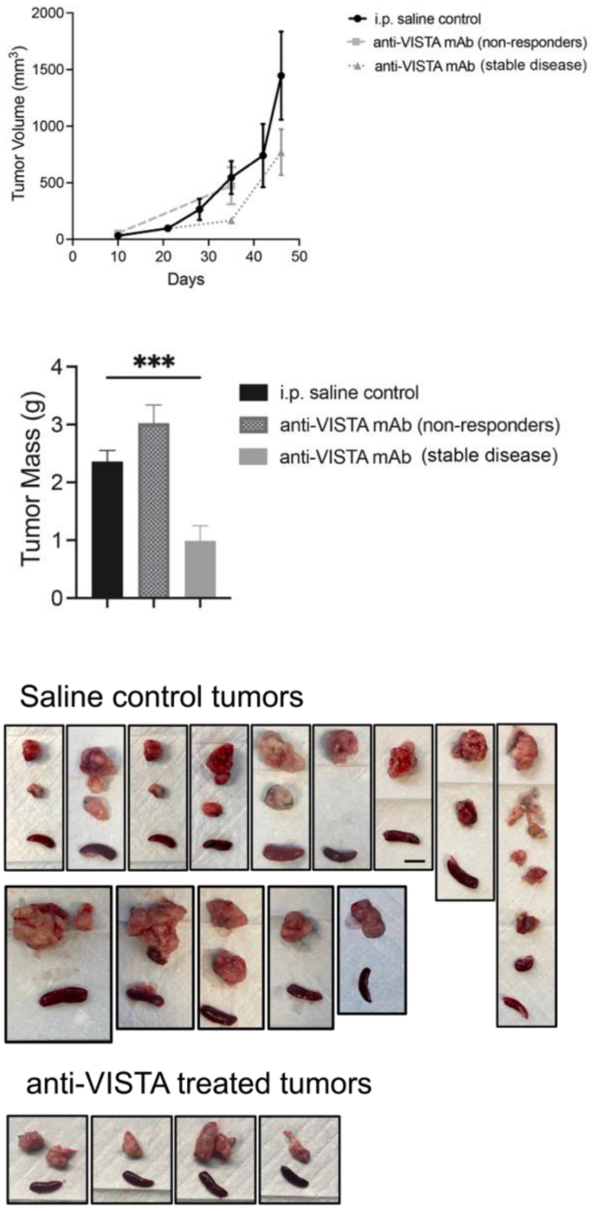

Figure S1. Longitudinal tumor growth in anti-VISTA mAb tumors (and corresponding controls) quantified from serial MT218-MRMI and T_2-weighted MRI. Three-dimensional ROI analysis was employed to extract tumor volumes of saline controls and anti-VISTA mAb-treated mice (non-responders and stable disease). Following the final MRMI scan, mice were euthanized and tumors and spleens were excised. Tissues were weighed and imaged to verify MRMI findings. Tissues were saved for further histopathological assessment. Bar = 1 mm; ***$p < 0.001$.

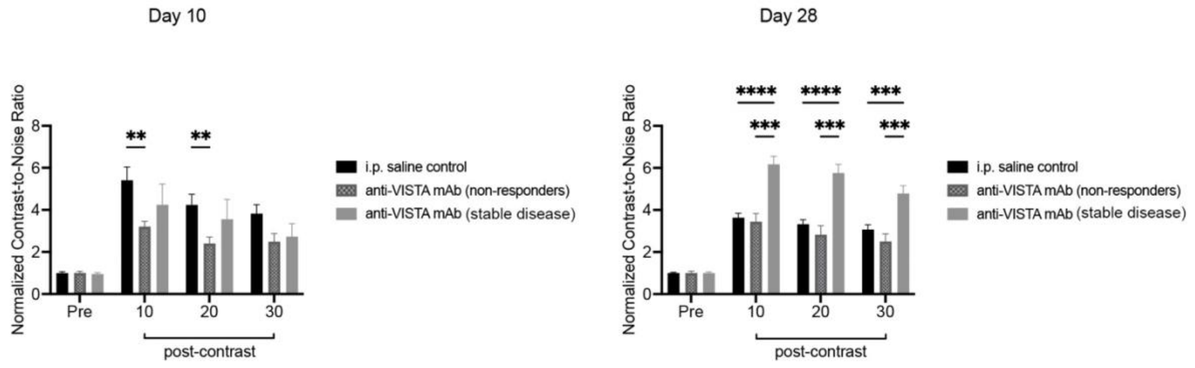

Figure S2. Mean normalized CNR ratios from anti-VISTA-treated mice and associated control group. ROI analysis was conducted on anti-VISTA-treated tumors (non-responders (n = 6) and stable disease (n = 8)) and controls (n = 15). As most mice in both groups expired before day 46, there was an insufficient sample size for statistical analysis. *p < 0.05**.**

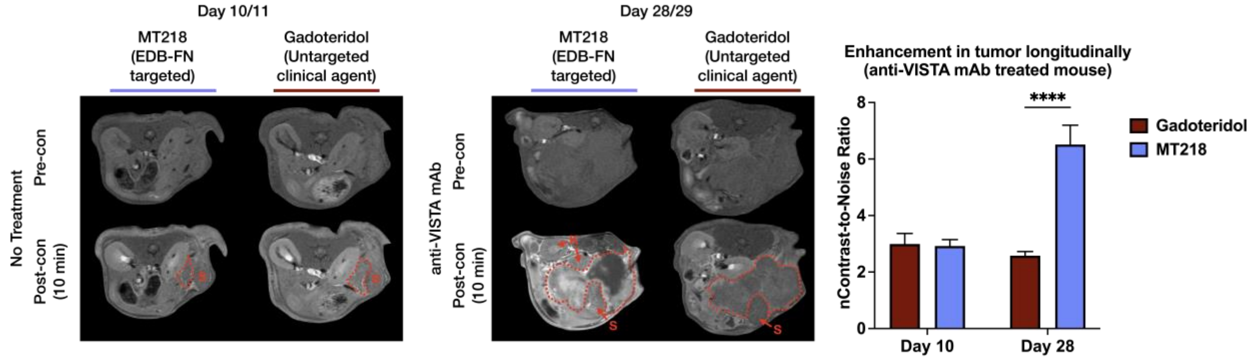

Figure S3. Comparison of MT218-MRMI tumor enhancement with untargeted clinical agent, gadoteridol, with and without anti-VISTA therapy. Tumors were imaged prior to treatment on day 10/11 and again on day 28/29 following 6 doses of anti-VISTA therapy. Tumor enhancement (10 min post-contrast) was quantified using normalized CNR. Tumor boundaries are illustrated via red dashed lines. Spleens (S) and kidneys (K) are labeled and identified with red arrows where appropriate. ****p < 0.0001.

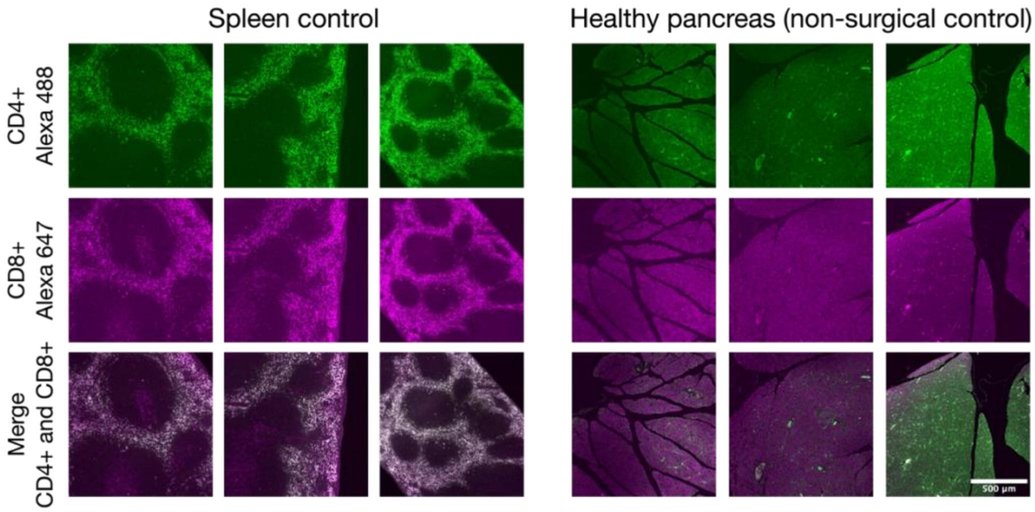

Figure S4. Additional immunofluorescence (IF) staining of normal murine spleens and pancreata. Tissues were dual stained for $CD4^+$ (Alexa 488, green) and $CD8^+$ (Alexa 647, magenta) surface markers.

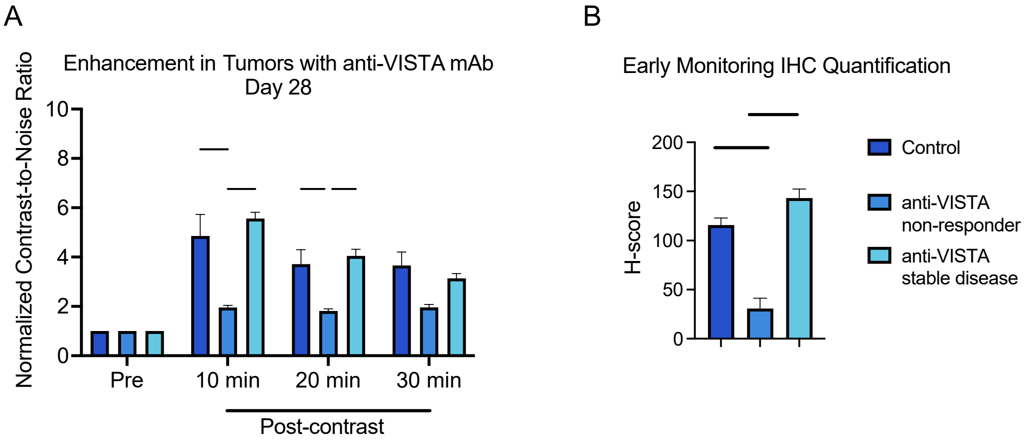

Figure S5: **High MRMI contrast-to-noise ratio (CNR) and signal intensity in treated tumors with MT218 contrast enhancement correlating with IHC staining of EDB-FN on day 28. A)** Normalized CNR of tumors excised at earlier time point. **B)** Quantification of immunochemical anti-G4 staining of EDB-FN. Contrast-enhanced MRI signal intensity was correlated with EDB-FN expression determined by IHC staining (H-score) in corresponding treatment groups.

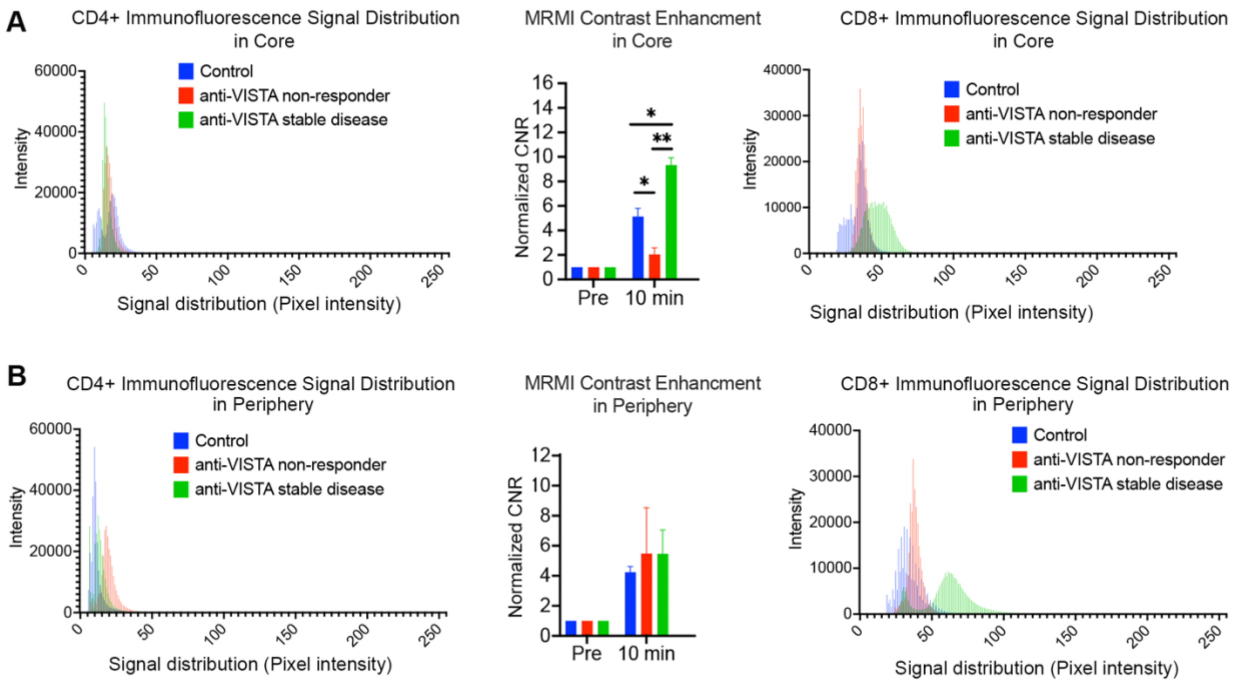

Figure S6. CD4$^+$ and CD8$^+$ IF signal distribution within tumor core and corresponding MRMI signal at 10 min post-contrast within the same core region (A) vs from the "periphery" (B) of the tumor tissues treated with anti-VISTA mAb. Quantitative assessment of T cell staining in Figure 3, histograms display signal intensity (x-axis) versus pixel frequency (y-axis) across three groups: control, anti-VISTA non-responders, and anti-VISTA stable disease mice. The tumor core regions (A) from stable disease mice showed significantly stronger CD4$^+$ staining compared to non-responders and controls, correlating directly with MRI CNR measurements at 10 minutes post-contrast. Conversely, tumor periphery regions (B) demonstrated no significant differences in CD4$^+$ staining between treatment groups, consistent with corresponding MRI findings in these areas. The abundance of CD8$^+$ T cells in anti-VISTA stable disease mice was reduced compared to other groups, indicating that CD8$^+$ T cells were less abundant at the tumor periphery in stable disease mice. Nevertheless, the anti-VISTA treatment did not result in significant survival improvement as compared to vaccine treatment.

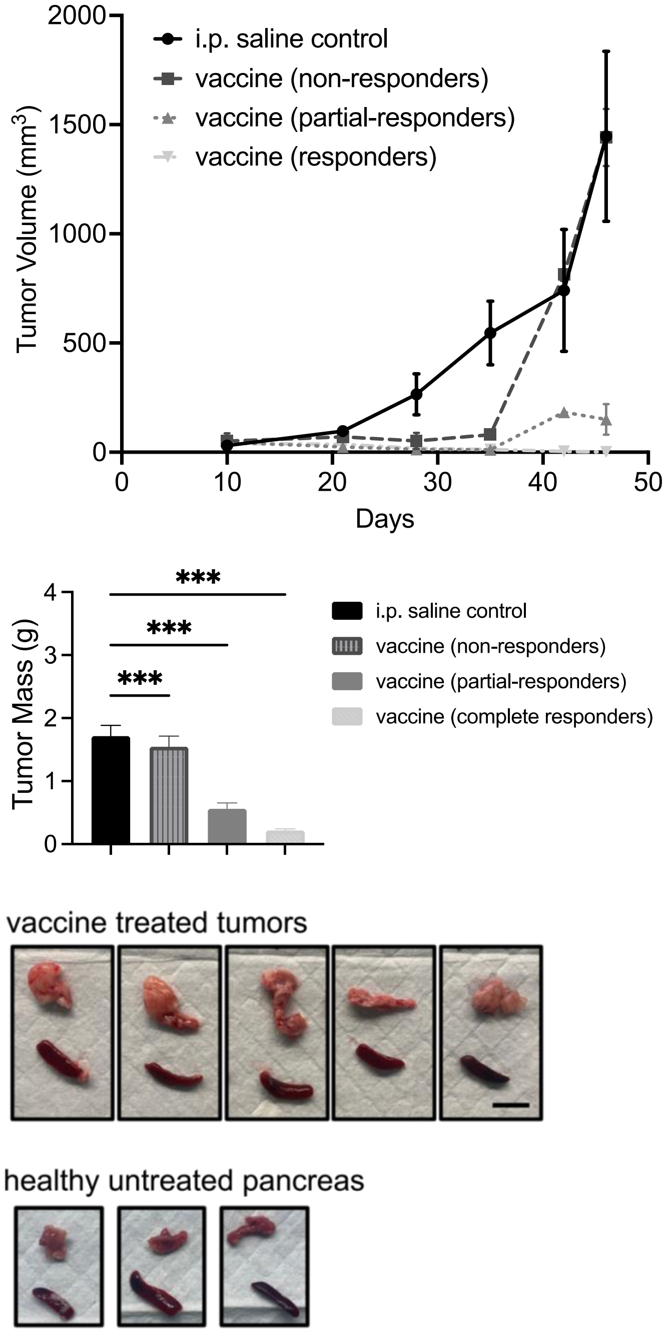

Fig. S7. Tumor growth of vaccine-treated mice quantified from serial MT218-MRMI and non-contrast T_2-weighted MRI. ROI based analysis was conducted at discretized time points for vaccine-treated mice and their associated controls. Tumors were excised from mice following the final MRMI scan. Tumors, pancreatic tissues and spleens were weighed and imaged for verification of MRMI findings. Normal pancreas, untreated, are also shown for reference. Tissues were saved for further histopathological assessment. Bar = 1 mm; ***$p < 0.001$.

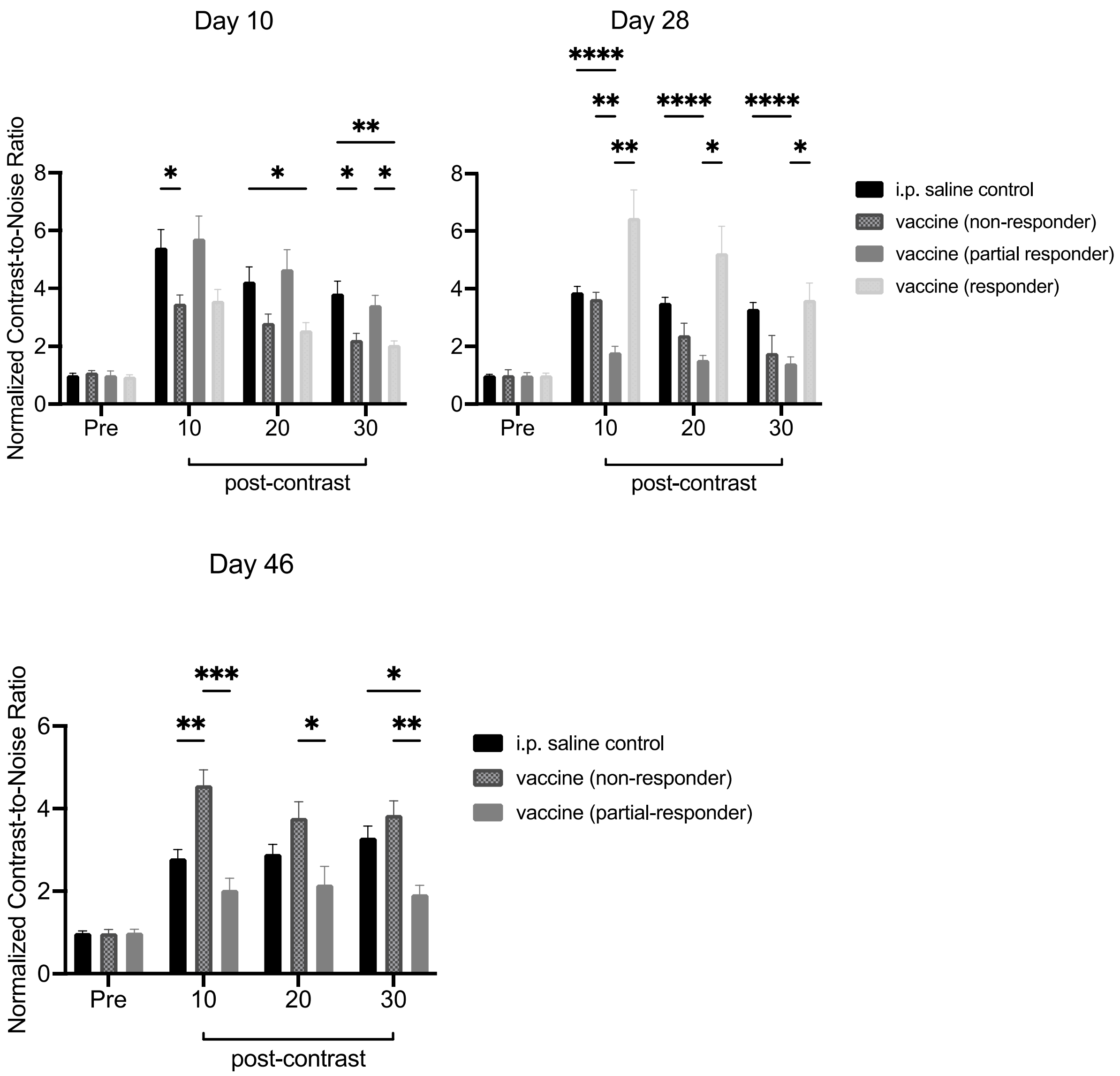

Fig. S8. Mean contrast-to-noise ratios (CNR) were determined for tumors in mice treated with vaccine or their associated controls. ROI analysis of tumor regions was done on control tumors (n = 21) and vaccine tumors: non-responders (n = 9), partial responders (n = 10), and tumor-free complete responders (n = 11). Vaccine-treated mice with tumor-free outcomes could not be measured at day 46 due to absence of tumor and negligible contrast accumulation in the pancreas. *$p < 0.05$, **$p < 0.01$, ***$p < 0.001$.

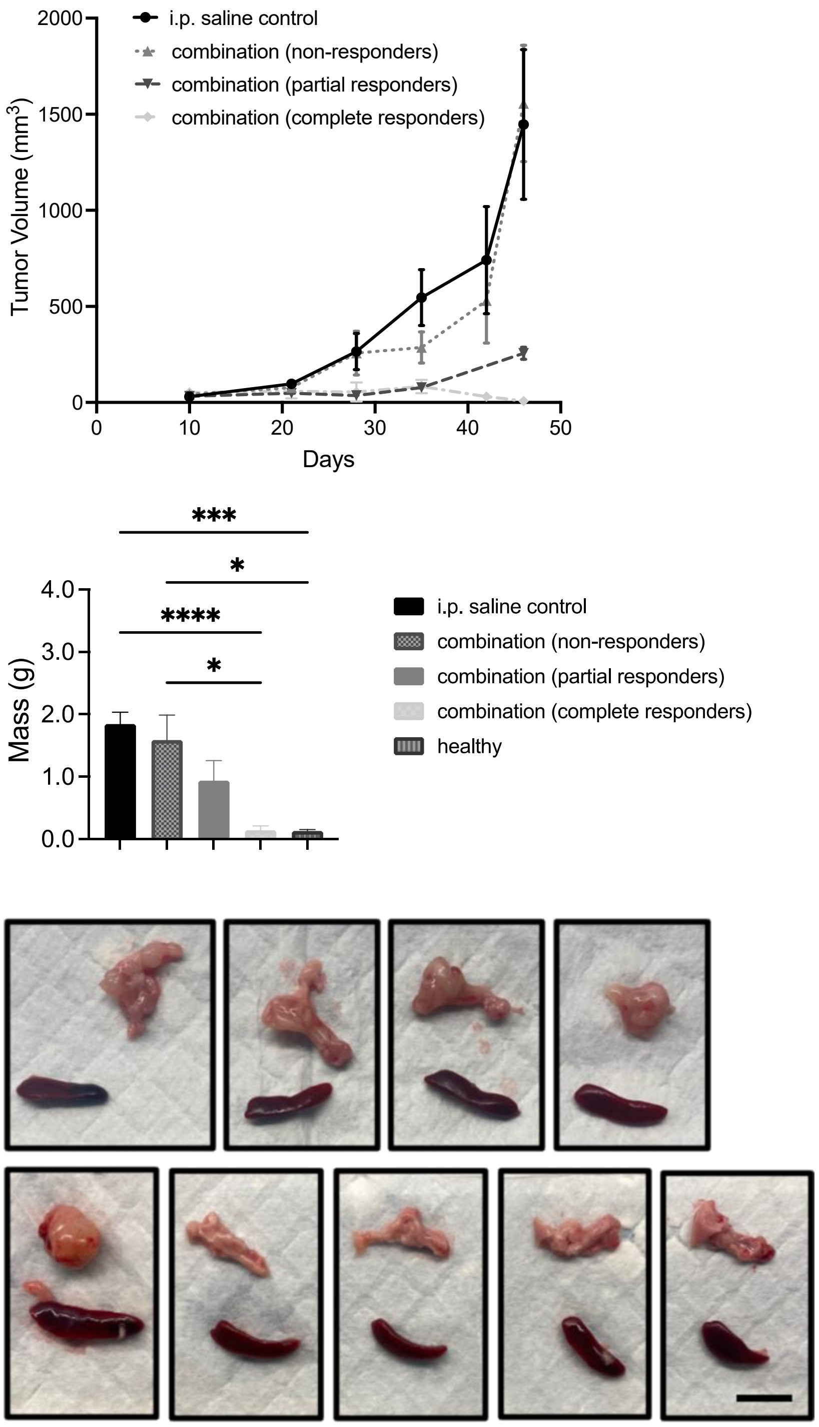

Fig. S9. Tumor volumes quantified through ROI analysis on MT218-MRMI and non-contrast T_2-weighted MRI. 3D volumes were calculated at various time points for mice treated with combination therapy (non-responders, partial responders and complete responders). Tumors, pancreatic tissues and spleens were excised following the final MRMI scan and tumor masses were weighed and imaged for use as verification of MRMI findings. Bar = 1 mm; *$p < 0.05$, **$p < 0.01$, ***$p < 0.001$, ****$p < 0.0001$.

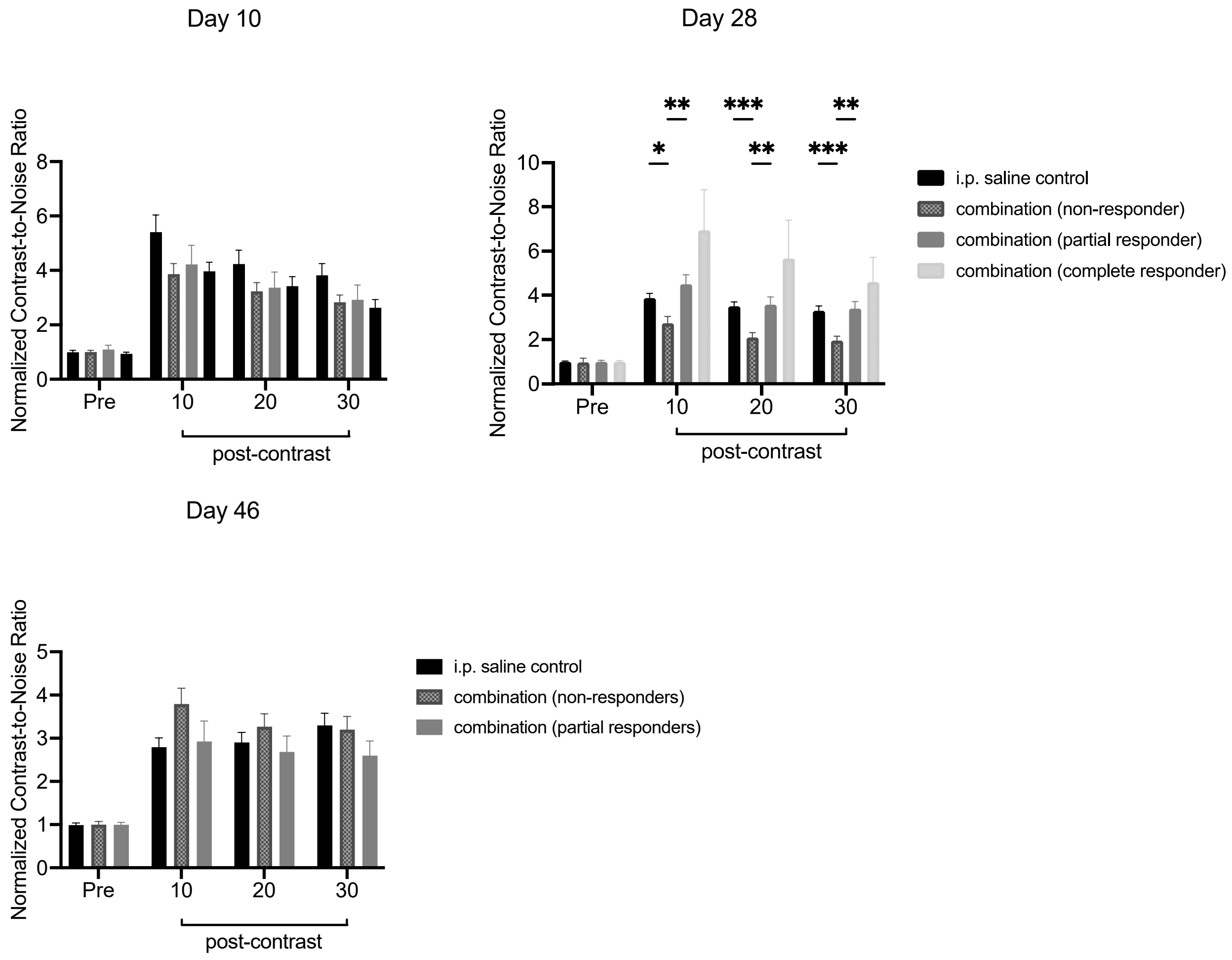

Fig. S10. Mean contrast-to-noise ratios (CNR) in the tumors of the mice treated with saline controls (n = 28) and non-responders (n = 4), partial responders (n = 5), and complete responders (n = 13) treated with the combination of the vaccine cocktail and anti-VISTA mAb. Bar = 1 mm; *p < 0.05, p < **0.01, ***p < 0.001, ****p < 0.0001
